# Regulation of Excitonic Behavior in Defective Acetylenic Polymers Enables Mechanism Switching in O_2_ Activation for Enhanced Water Decontamination

**DOI:** 10.1002/advs.202511534

**Published:** 2025-12-17

**Authors:** Xiaofeng Tang, Sijia Jin, Wei Li, Yingrong Wang, Haiyan Zhang, Zhiqiao He, Shuang Song, Yaqi Cai, Tao Zeng

**Affiliations:** ^1^ Key Laboratory of Microbial Technology for Industrial Pollution Control of Zhejiang Province Department of Environment Zhejiang University of Technology Hangzhou Zhejiang 310032 P. R. China; ^2^ Zhejiang Key Laboratory of Environment and Health of New Pollutants School of Environment Hangzhou Institute for Advanced Study University of Chinese Academy of Sciences Hangzhou 310024 P. R. China; ^3^ State Key Laboratory of Environmental Chemistry and Ecotoxicolog Chinese Academy of Sciences Beijing 100085 P. R. China; ^4^ Shaoxing Research Institute Zhejiang University of Technology Shaoxing Zhejiang 312000 P. R. China

**Keywords:** defective acetylenic polymers, exciton behavior manipulation, O2 activation pathway, orientated charge accumulation, theoretical guidance

## Abstract

Understanding the microenvironment structure−activity relationship of photo‐responsive polymers is crucial to steer the charge carrier flow for molecular oxygen (O_2_) activation. However, the spin‐forbidden nature of O_2_ and the inherent Frenkel exciton effect hinder the efficient O_2_ activation, particularly in achieving selective reactive oxygen species (ROSs) generation. Herein, the Sabatier volcano plot is first utilized to manipulate the microenvironment of poly(1,3,5‐triethynylbenzene) (PTEB) via molecular defect‐mediated charge accumulation to regulate the exciton behavior. The screened PTEB‐CN and PTEB‐NH_2_, with thermodynamic advantages, are created artificial internal electric field (IEF) to induce exciton dissociation and oriented migration. Meanwhile, the significant weakening of exciton binding energy (E_b_) in defective PTEBs overcomes the Frenkel exciton effect, switching the O_2_ activation from a traditional energy transfer‐mediated nonradical route (pristine PTEB) to a hot charge‐driven radical pathway. Mechanism inquiry reveals the reversely oriented IEF dictates the migration direction of charge carriers, leading to predominant migration of photo‐induced electron (e^−^) in conjugated sites toward the ─NH_2_ defect, while ─CN defect is primary occupied with photo‐induced hole (h^+^). The polarized distribution of charge carriers in PTEB‐NH_2_ endows the polymeric semiconductor with enhanced selectivity for superoxide radical (O_2_^•−^) generation and improved contaminant removal efficiency. This work offers promising prospective for regulating exciton behavior for organic polymers and opens a frontier for O_2_ activation.

## Graphical abstract

A molecular defect‐mediated charge accumulation strategy is developed to regulate the exciton behavior for selective molecular oxygen (O_2_)activation in poly(1,3,5‐triethynylbenzene) (PTEB). Artificial internal electric field (IEF) constructed in defective PTEB provides internal driving force to steer the directed migration of charge carriers, thereby switching the O_2_ activation from traditional energy transfer (primarily yielding singlet oxygen (^1^O_2_)) to a hot charge‐driven pathway (primarily yielding superoxide radical (O_2_^•−^)).

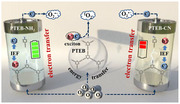

## 1 Introduction

**Figure 1:**
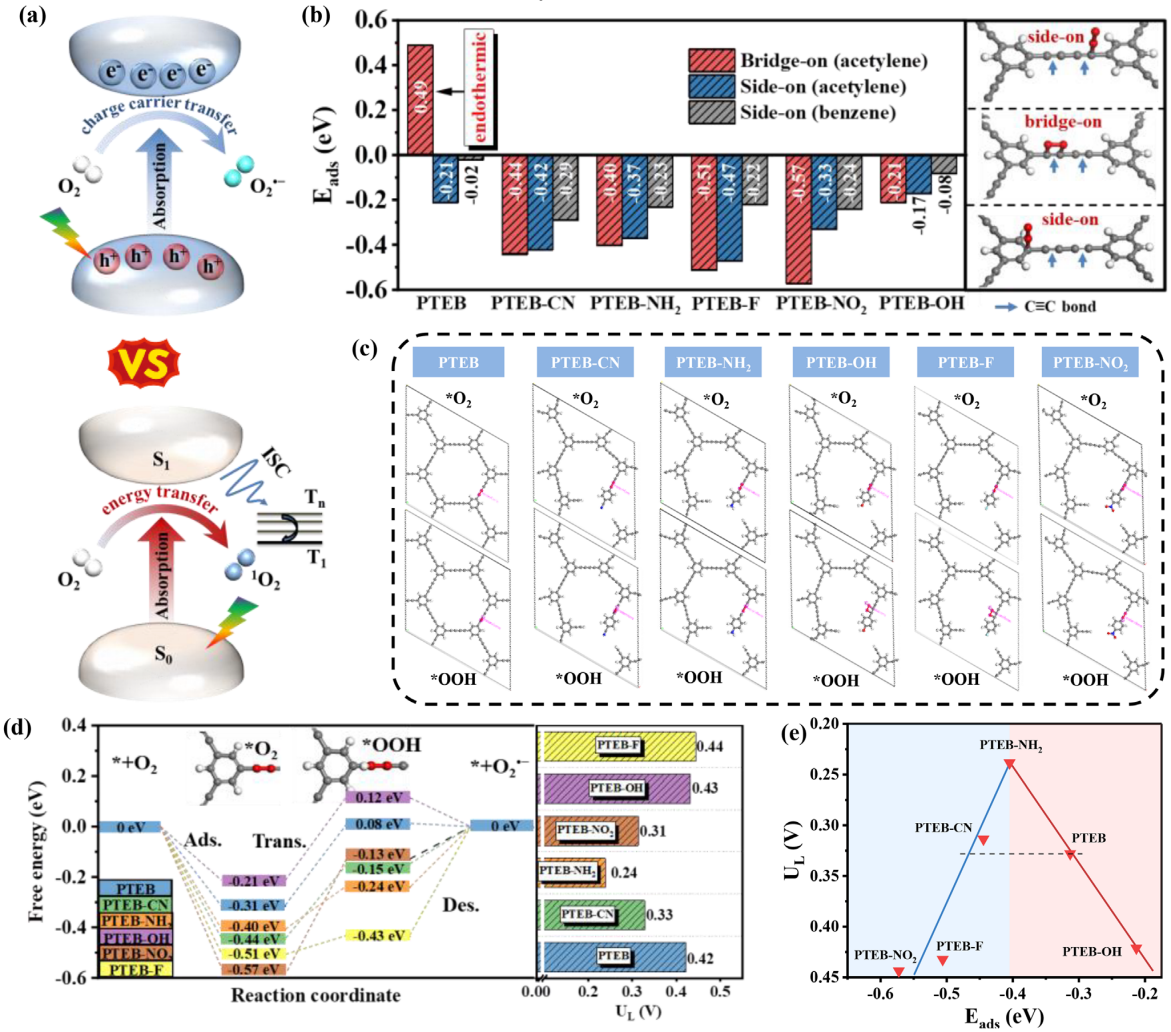
a). Illustration of the O_2_ activation involving charge‐carrier transfer and energy transfer pathways. b) Calculated adsorption energy for different adsorption configurations. c) Polymeric structures representing different possible O_2_ adsorption configurations. d) Potential energy profiles of the reaction pathway for O_2_ activation. e) Activity volcano plots showing the calculated limiting potential.

The advanced oxidation processes (AOPs) based on molecular oxygen (O_2_) activation are a novel technique with environmental beginning prospect owing to the naturally occurring O_2_ in the atmosphere.^[^ ^1^, ^2^ ^]^ Yet the naturally occurring ground‐state O_2_ (^3^O_2_) encounters a spin‐forbidden reaction due to the parallel spin orientation of its two outermost electrons in degenerate π* antibonding orbitals,^[^ ^3^, ^4^ ^]^ which impedes O_2_ activation under mild conditions due to its relative stable structure. To weaken the O─O bond and overcome the thermodynamic barrier, employing metal‐free, visible‐light‐responsive conjugated polymers offers an effective and environmentally friendly strategy for molecular oxygen activation as it avoids secondary pollution from metal ions and makes use of renewable solar energy.^[^ ^5^, ^6^, ^7^ ^]^ Molecular O_2_ can typically be activated and transformed into reactive oxygen species (ROS) through either charge‐carrier transfer pathways or exciton transfer pathways. Generally, charge carrier transfer through exciton dissociation typically accounts for the production of radical species (O_2_^•−^ and •OH), while energy transfer involving singlet excitons transitioning to triplet excitons via intersystem crossing (ISC) primarily leads to the generation of non‐radical species (^1^O_2_) (**Figure** **1**a).^[^ ^8^, ^9^ ^]^ However, due to the inherent strong exciton binding energy (E_b_, typically>100 meV)^[^ ^8^ ^]^ caused Frenkel exciton effect,^[^ ^10^ ^]^ conjugated organic polymers predominantly rely on exciton transfer pathways to generate nonradical species,^[^ ^11^, ^12^ ^]^ while relevant studies concentrate on conjugated organic photocatalyst mediated O_2_ activation through charge‐carrier transfer pathway for radical generation remain underdeveloped. As a matter of fact, free radicals are typically characterized by significantly higher oxidative potential (1.9–3.1 V) compared to non‐radical species (0.6–1.2 V),^[^ ^9^, ^13^ ^]^ which is beneficial for contaminant degradation and mineralization. The essential factor for the occurrence of the charge‐carrier transfer pathway lies in the dissociation of excitons into hot e^−^ or h⁺, rather than their persistence as electron‐hole pairs, thereby promoting charge carrier transfer and suppressing energy transfer. Up to date, advances in exciton dissociation steering have received limited success because the most commonly used strategies are overwhelmingly focused on metal or metal oxides‐based inorganic heterojunctions or the loading of metal atoms onto organic substrates.^[^ ^14^, ^15^, ^16^, ^17^ ^]^ However, these conventional strategies involving inorganic metal ions entail complex procedures and predominantly affect interface exciton behavior without significantly influencing bulk charge separation, making them challenging to apply to conjugated polymeric materials.

Artificially constructing the internal electric field (IEF) within the conjugated polymeric structure would be an effective solution, as it provides an overall internal driving force to overcome strong E_b_, thereby facilitating oriented separation of bulk charge carriers.^[^ ^10^ ^]^ Conjugated polymers constructed by vastly available building blocks become a versatile platform to achieve IEF construction through rational choice of linker molecules and defined defect precursors (“bricks”), enables the reasonable designing of targeted polymeric structure with regulated charge accumulation.^[^ ^18^, ^19^ ^]^ Among the constructive strategies, defect engineering via “bricks” substitution has been reported as an effective method for manipulating the IEF at the molecular level, as it can regulate the electronic band structure, leading to a nonuniform local charge distribution, steer charge flow in O_2_ activation, and even switch the reaction pathway.^[^ ^20^ ^]^

Another critical factor influencing the charge‐carrier transfer‐mediated O_2_ activation performance is the binding dynamics between molecular O_2_ and the polymeric structure. Owing to the strong affinity between acetylene moieties and peroxide bonds,^[^ ^21^ ^]^ conjugated acetylenic polymers (CAPs) with surface‐rich diacetylenic linkers could achieve continuous adsorption and dissociation of molecular O_2_.^[^ ^22^, ^23^, ^24^ ^]^ Therefore, intentional selection of acetylenic “building blocks” and deliberate breaking of certain bricks of the polymeric framework enables controlled terminal defects, achieving artificial construction of localized IEF and simultaneously facilitating O_2_ binding affinity. Theoretically, while a diverse array of defective CAPs can be synthesized via an infinite selection of “blocks” to create artificial defects, as a matter of fact, the resulting materials commonly possess considerable variability in photocatalytic activity owing to their discriminative properties. In this context, theoretical predictions offer an efficient strategy for screen out suitable structural defects in constructing CAPs framework, minimizing experimental trial‐and‐error processes and thereby saving both time and economic resources.

Herein, based on the two key steps of adsorption and product transformation in the O_2_ activation process, we utilized density functional theory (DFT) calculations to thermodynamically evaluate the effect of various structural defects on modifying the overpotential for O_2_^•−^ generation over a series of different functional defects based on the model of poly(1,3,5‐triethynylbenzene) (PTEB). To rationally establish an IEF within the polymeric framework, representative functional groups exhibiting strong electron‐withdrawing or electron‐donating characteristics, namely ─CN, ─NH_2_, ─F, ─NO_2_ and –OH were deliberately introduced as terminal defects to precisely modulate the electronic distribution across the π‐conjugated plane of the polymer. The incorporated these terminal defects in polymeric structure to partially replace partial diacetylenic linkers is revealed to switch the O_2_ adsorption configuration from side‐on type to bridge‐on type, among which the –NH_2_ and –CN defects are able to effectively lower the potential barrier for O_2_^•−^ generation. Following the calculated results, the target polymers with terminal–NH_2_ and –CN defects (PTEB‐NH_2_ and PTEB‐CN) are synthesized. As expected, the incorporated functional defects were able to regulate the local electronic distribution, thereby constructing IEF, under which the hot excitons tend to dissociate into charge carriers (e^−^ and h^+^). As such, the photocatalytic O_2_ activation mechanism successfully switched from energy transfer pathway (pristine PTEB) to charge carrier transfer pathway (PTEB‐NH_2_ and PTEB‐CN), different functional defects lead to varying catalytic effects. Experimental results and theoretical prediction reveal that the incorporation of ‐NH_2_ defects has the capability to reduce the binding energy of hot excitons, facilitating the oriented migration of hot e^−^ toward the adjacent acetylenic sites where O_2_ is adsorbed. Diverging from the process in pristine PTEB, where hot excitons generate ^1^O_2_ through the ISC process, this charge‐carrier transfer pathway not only alters the thermodynamics of ROS generation but also impacts the subsequent kinetics of pollutant removal, making it a radical‐driven, highly efficient degradation system.

## 2 Results and Discussion

### 2.1 Designing Optimal π‐Conjugation Environment of PTEB Guided by Theoretical Calculations

Generally, O_2_ can be adsorbed onto a single active site with side‐on form to generate a side‐on configuration. However, pioneer research uncovered an thermodynamic‐favored bridge‐on configuration of acetylene moieties for capturing O_2_ molecules.^[^ ^21^ ^]^ To understand the role of functional defects and guide the polymeric microenvironment design, six different configurations with acetylenic monomers partially replacement by –CN, –NH_2_, –F, –NO_2_ and –OH defects, were systematically evaluated for the potential in constructing PTEBs (Figure S1, Supporting Information). Various O_2_ adsorption configurations with both side‐on and bridge‐on types were included to identify the primary active sites (Figure S2, Supporting Information). The results showed that the adsorption energies (E_ads_) of O_2_ on PTEB were determined to be −0.02 and −0.21 eV for side‐on adsorption on benzene rings and acetylene moieties (Figure 1b), respectively. While O_2_ encounters an energy barrier of 0.49 eV for adsorption in the bridge‐on configuration on the acetylene moieties of PTEB. These results suggest that side‐on adsorption of O_2_ on the acetylene moieties is a predominant adsorption configuration on PTEB. Upon the introduction of –CN, –NH_2_, –F, –NO_2_, and –OH defects, the bridge‐on configuration is liberated from the constraints of the energy barrier, rendering it a thermodynamically spontaneous process, with E_ads_ of −0.44, −0.40, −0.51, −0,57 and −0.21 eV, respectively. Comparatively, the calculated E_ads_ of the side‐on adsorption configuration reveal significantly lower energy, indicating that the bridge‐on form is the predominant adsorption configuration for these defective PTEB.

The introduction of the terminal defects uniformly demonstrates a significant enhancement in the thermodynamics of O_2_ adsorption, given the comparative analysis of their respective optimal E_ads_ surpasses that of pristine PTEB. However, excessively strong intensity of the reactants may hinder the formation of critical intermediates in the charge carrier transfer pathway due to active site poisoning, thereby reducing the generation of radical species.^[^ ^25^, ^26^ ^]^ In the context of O_2_ activation, protonated O_2_^•−^ (OOH) serves as a key intermediate for the generation of various radical species via the charge carrier transfer pathway (Equations (1), (2), (3)).^[^ ^27^, ^28^ ^]^

(1) $$O_{2}+e^{-}\;\to \;{O_{2}}^{•}$$

(2) $${O_{2}}^{•-}+e^{-}+2H^{+}\to H_{2}O_{2}$$

(3) $$H_{2}O_{2}+e^{-}\to •OH+OH^{-}$$

Thus, achieving optimal radical species‐dominated O_2_ activation process requires a delicate balance between O_2_ adsorption strength and limiting potential in O_2_^•−^ generation according to the Sabatier relation.^[^ ^29^, ^30^ ^]^ According to the calculation result (Figure 1c,d), molecular O_2_ activation upon the different photocatalyst models, the endothermic reaction primarily occurs on the generation of O_2_^•−^ precursor (OOH) or the desorption process of O_2_^•−^. The calculated limiting potentials for different stages of reactant‐catalyst composite models during the stepwise reaction of pristine PTEB were 0.33 V, with the rate‐determining step (RDS) primarily identified as the transformation of adsorbed *OO into adsorbed *OOH. Using the E_ads_ of O_2_ as the descriptor, the theoretical Sabatier volcano plot is displayed in Figure 1e. The upper limit of the reaction activity, as depicted in the volcano plot, is governed by the formation energy of OOH and the desorption energy of O_2_^•−^. Among all the samples, PTEB‐CN and PTEB‐NH_2_ are the only two catalysts that are closer to the vertex of the volcano plot compared to pristine PTEB, with limiting potentials of 0.31 and 0.24 V, respectively. Accordingly, the incorporated –CN and –NH_2_ defects synergistically cooperates with active site, diacetylenic moiety, to reduce O_2_^•−^ formation overpotential, thereby providing a thermodynamic advantage for the charge carrier transfer pathway.

### 2.2 Synthesis and Structural Characterization of PTEB and d‐PTEB

**Figure 2:**
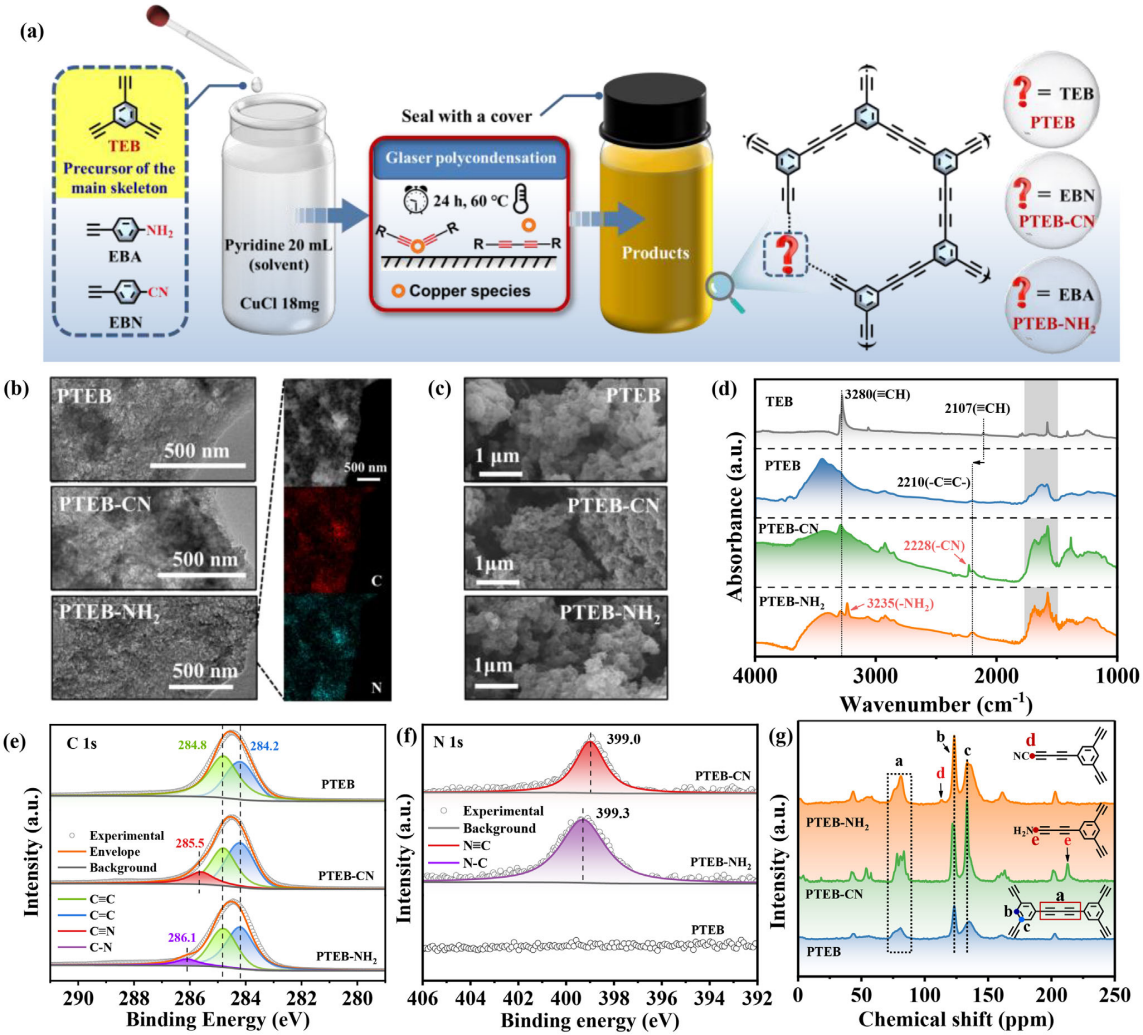
a) Schematic diagram for preparation of PTEB, PTEB‐NH_2_, and PTEB‐CN. b) TEM images of PTEB, PTEB‐CN, and PTEB‐NH_2,_ and EDS image of PTEB‐NH_2_. c) SEM image and photograph of PTEB, PTEB‐CN, and PTEB‐NH_2_. d) FTIR spectra of TEB, PTEB, PTEB‐NH_2,_ and PTEB‐CN. e) XPS C1s spectra of PTEB, PTEB‐NH_2,_ and PTEB‐CN. f) XPS N1s spectra of PTEB, PTEB‐CN, and PTEB‐NH_2_. g) Solid‐state ^13^C NMR spectra of PTEB, PTEB‐NH_2,_ and PTEB‐CN.

According to the guidance from DFT calculations, photocatalysts with specific molecular structures should be precisely synthesized. Here, PTEB, PTEB‐CN, and PTEB‐NH_2_ were synthesized through a typical catalytic Glaser coupling of acetylenic monomers using copper (I) salts as the catalyst (**Figure** **2**a, details were provided in Supporting Information). After the synthesis reaction, the powdered PTEB (brilliant yellow), PTEB‐CN (brown), and PTEB‐NH_2_ (isabellinus) were obtained (Figure S3, Supporting Information). Visualization by transmission electron microscopy (TEM) indicates the sheet‐like morphology of the synthesized PTEB, PTEB‐CN, and PTEB‐NH_2_ (Figures 2b; S4–S6, Supporting Information). High‐resolution transmission electron microscopy (HRTEM) reveals the typical characteristics of amorphous polymers, without any observable lattice fringes (Figure S7, Supporting Information). Moreover, the selected‐area electron diffraction (SAED) patterns show no distinct diffraction rings, indicating the absence of crystallographic planes in PTEB, PTEB‐CN, and PTEB‐NH_2_, further confirming their amorphous polymer structures (Figure S8, Supporting Information).

TEM‐related energy‐dispersive X‐ray spectroscopy (EDX) mappings of PTEB‐NH_2_ and PTEB‐CN illustrate that the N element was uniformly distributed over the whole polymeric structure (Figure S9, Supporting Information), indicating the successful introduction of –NH_2_ and –CN defects after the Glaser polycondensation. The field emission scanning electron microscope (FE‐SEM) images revealed the coral‐like morphology of the collected samples (Figure 2c), which facilitates the exposure of more active sites and thus promotes the catalytic O_2_ activation process. Additionally, the structure of polymeric samples could be confirmed by Fourier transform infrared spectroscopy (FTIR) analysis. As shown in Figure 2d, the peaks located at 2107 and 3280 cm^−1^ could be attributed to the typical stretching modes of terminal C≡C bonds of TEB. After Glaser polycondensation, the FTIR spectra of PTEB show a significant reduction in the peaks associated with terminal C≡C moieties due to C–C coupling of these groups, with the peaks nearly disappearing. However, it is noted that the FTIR spectra of PTEB‐CN and PTEB‐NH_2_ still display the peaks of terminal C≡C bonds. This is attributed to the existing terminal acetylene groups due to the incorporated artificial defects, which suppress the complete polymerization and allow some terminal acetylene groups to remain unpolymerized, indirectly indicating the formation of –CN and –NH_2_ defects. The newly emerged IR peaks at 3235 cm^−1^ of PTEB‐NH_2_ can be attributed to the characteristic amino stretching vibration due to the incorporation of –NH_2_ defects.^[^ ^31^ ^]^ Similarly, the terminal nitrile stretching vibration at 2228 cm^−1^ observed in the FTIR spectra of PTEB‐CN confirms the successful incorporation of –CN defects.^[^ ^32^, ^33^ ^]^ The formation of diacetylenic bonds is proved by the characteristic peaks at 2210 cm^−1^.^[^ ^22^, ^34^ ^]^ And the adsorption region located at 1585–1629 cm^−1^ was ascribed to the skeletal vibration of the aromatic ring.^[^ ^35^ ^]^ Meanwhile, the C 1s X‐ray photoelectron spectroscopy (XPS) spectra of PTEB can be deconvoluted into two subpeaks located at 284.2 and 284.8 eV, corresponding to C═C (sp^2^) and C≡C (sp) hybridized carbons (Figure 2e), respectively.^[^ ^18^ ^]^ New emerging peakS at 285.5 and 286.1 eV, assigned to the C≡N and C─N bonds, respectively,^[^ ^36^ ^]^ substantiating the successful incorporation of –CN and–NH_2_ defects within the polymeric structure of PTEB‐CN and PTEB‐NH_2_. This finding was further proved by N 1s XPS spectra, in which the PTEB‐CN and PTEB‐NH_2_ presented peaks at 399.0 and 399.3 eV assigned to N≡C and N─C bonds, respectively, as the –CN and –NH_2_ defects incorporated (Figure 2f).^[^ ^32^, ^37^, ^38^ ^]^ The solid‐state ^13^C nuclear magnetic resonance (NMR) spectra (Figure 2g) provide detailed information for polymeric skeletons, in which the peaks at 81.5 ppm could be assigned to the C atoms in diacetylene moieties.^[^ ^18^, ^35^ ^]^ While the peaks located at 123.6 and 135.0 ppm are attributed to the meta‐carbons and para‐carbons of benzene ring with in the polymeric structure.^[^ ^18^, ^35^ ^]^ Compare with PTEB, new emerging peaks at 215.1 and 113.5 ppm could be assigned to the C atoms in the terminal CN groups and C‐NH_2_ bonds of PTEB‐CN and PTEB‐NH_2_, respectively, which illustrate that the target polymers equipped with different terminal defects were successfully synthesized via Glaser coupling reaction.^[^ ^39^, ^40^ ^]^

### 2.3 Photocatalytic O_2_ Activation for Contaminant Degradation

**Figure 3:**
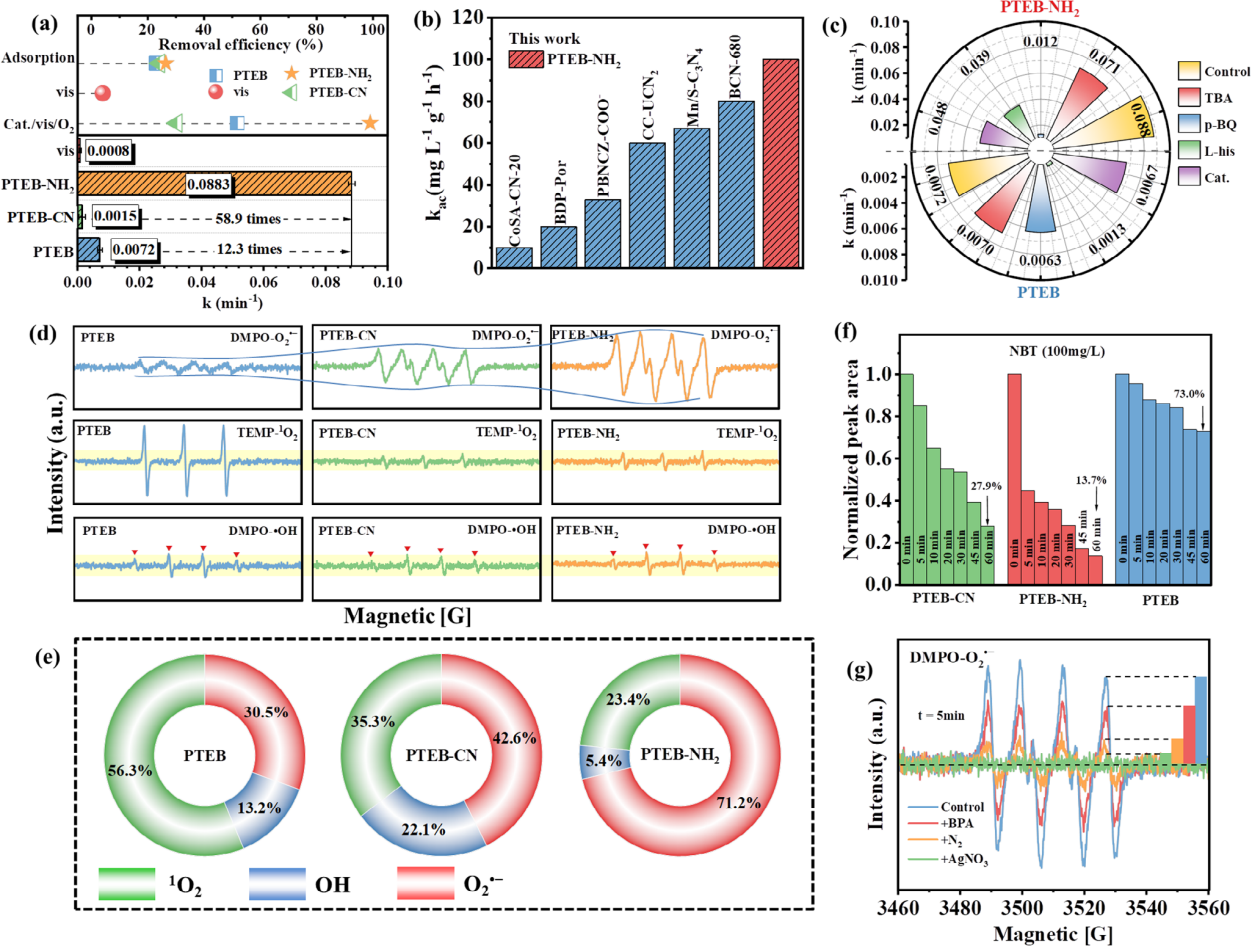
a) BPA removal efficiency and reaction constant of different systems. b) The specific O_2_ activation activity of PTEB‐NH_2_ compared to other state‐of‐the‐art organic polymers. c) Effect of scavengers during the degradation of BPA in different systems. d) EPR spectra for DMPO‐•OH, DMPO‐O_2_^•−^ and TEMP‐^1^O_2_ adducts detection in various catalytic systems. e) The calculated contribution of ROSs in each system. f) Integral normalized peak area of NBT in various systems. g) DMPO spin‐trapping spectra in the PTEB‐NH_2_/O_2_/vis system with/without BPA, N_2_ purging, and AgNO_3_.

The photocatalytic activities of all the samples were evaluated by degrading bisphenol A (BPA) under visible light (λ>400 nm) irradiation. For comparative analysis, the direct oxidation of BPA under visible light in the absence of a photocatalyst was evaluated under the same experimental conditions. As shown in **Figures** **3**a and S11 (Supporting Information), without the participation of the photocatalyst, visible light irradiation did not result in any notable removal of BPA. The control experiments also revealed that the obtained polymers exhibited moderate adsorption performance (≈25%), with adsorption−desorption equilibrium being established within ≈30 min (Figure S10, Supporting Information). Accordingly, subsequent contaminant degradation experiments were conducted after allowing 30 min for the adsorption−desorption equilibrium to be reached. Figure S11 (Supporting Information) presents that PTEB‐NH_2_ achieves ≈99% BPA removal within 60 min, significantly outperforming pristine PTEB, which achieves only ≈52% degradation efficiency under identical conditions. In stark contrast to the enhanced performance observed for PTEB‐NH_2_, PTEB‐CN exhibited significantly lower efficiency compared to pristine PTEB, achieving a BPA removal ratio of only 30.0%. This indicates that –CN and –NH_2_ defects exert distinct effects on for O_2_ activation. Comparative analysis of the reaction rate constants (k_obs_) reveals that PTEB‐NH_2_, PTEB, and PTEB‐CN possess k_obs_ values of 0.0883, 0.0072, and 0.0015 min^−1^ (Figure 3a), respectively. The rate of the PTEB‐NH_2_ is 12.3 times and 58.9 times higher than that of PTEB and PTEB‐CN, respectively, being one of the most active polymeric catalysts for photocatalytic molecular oxygen activation. Notably, the PTEB‐NH_2_ exhibits superior specific activity (k_ac_ = 100 mg L^−1^ g^−1^ h^−1^) compared to other state‐of‐the‐art organic photocatalysts, highlighting its superiority in the molecular O_2_ activation (Figures 3b; S12 and Table S1, Supporting Information), and maintain its catalytic stability under varying conditions (Figures S13 and S14, Supporting Information). The mineralization performance was first investigated by the determination of the total organic carbon (TOC) during the reaction. Nearly 57.4% of TOC was removed within 60 min by PTEB‐NH_2_/O_2_/vis system (Figure S15, Supporting Information), which significantly outperforms the BPA mineralization efficiency of the PTEB/O_2_/vis system (26.8%).To evaluate the contaminant mineralization pathway via PTEB‐NH_2_‐mediated O_2_ activation process, the Fukui function was applied (Table S2, Supporting Information), revealing that the C_2_, C_4_, C_12_, and C_14_ atoms of BPA are potential reactive sites. Combined with the results from liquid chromatography‐mass spectrometry (LC‐MS) (Figure S16, Supporting Information), degradation pathways involving hydroxylation and β‐scission were revealed (Figure S17, Supporting Information). On one hand, BPA can be attacked by O_2_^•−^ forming P5 through a hydroxylation pathway. Subsequently, P5 undergoes ring‐opening reactions to generate simple organic acids, which are ultimately mineralized into CO_2_ and H_2_O. On the other hand, BPA can also undergo electrophilic attack by O_2_^•−^ to produce P1 and P3, which similarly undergo ring‐opening reactions to form simple organic acids and are eventually mineralized into CO_2_ and H_2_O. The toxicity assessment of the corresponding intermediates reveals that the degradation process effectively reduces the biotoxicity of BPA (Figure S18 and Table S3, Supporting Information).

### 2.4 Terminal Defect Dependent Reaction Pathway Switching Mechanism

To investigate the primary ROSs involved in the BPA degradation process, radical quenching experiments were conducted. Initially, the N_2_ aeration experiment (Figure S19, Supporting Information) confirms that the O_2_ activation is the primary factor driving pollutant degradation in the PTEB‐NH_2_/O_2_/vis system. Quenching agents involving t‐butyl alcohol (TBA), p‐benzoquinone (p‐BQ), L‐histidine (L‐his), and catalase (CAT.) were employed as the scavengers for •OH, O_2_^•−^, ^1^O_2,_ and H_2_O_2_, respectively. For pristine PTEB, the addition of CAT. and TBA has a negligible effect on BPA degradation, whereas the injection of p‐BQ and L‐histidine resulted in inhibitory effects (Figure S20a, Supporting Information), suggesting that O_2_^•−^ and ^1^O_2_ are the primary reactive species accounting for O_2_ activation and subsequent BPA degradation. Comparative analysis of reaction constant reveals the inhibitory effect of L‐histidine is significantly stronger than that of p‐BQ, with the k_obs_ decreasing from 0.0072 to 0.0063 and 0.0013 min^−1^, respectively (Figure 3c), suggesting the PTEB/vis/O_2_ system is primarily mediated by ^1^O_2_. In PTEB‐NH_2_/vis/O_2_ system, a slight inhibitory effect of CAT. and TBA was observed, which provides evidence for the presence of H_2_O_2_ and •OH, while they do not take a leading role (Figures 3c; S20b, Supporting Information). However, the inhibitory effect of p‐BQ on BPA elimination is significantly stronger than that of L‐histidine, suggesting O_2_^•−^ is the main ROS produced in PTEB‐NH_2_/vis/O_2_ system. The prominent role of O_2_^•−^ is further proved by a quench experiment employing superoxide dismutase (SOD) as a quench agent, which suggests the distinction in the dominant ROS generated during the activation of O_2_ by PTEB and PTEB‐NH_2_ (Figure S20c, Supporting Information).

To further distinguish the ROSs, electron paramagnetic resonance (EPR) spectroscopy has been employed (Figure 3d). The 5,5‐dimethyl‐1‐pyrroline‐N‐oxide (DMPO) and 2,2,6,6‐tetramethylpiperidine (TEMP) were employed as radical trapping agents for identifying radical species (•OH and O_2_^•−^) and ^1^O_2_, respectively. It is noted that all the reaction systems exhibited rather weak EPR signals of DMPO‐•OH adducts, indicating the limited generation of •OH in all the systems. In the PTEB‐CN/O_2_/vis system, only very weak EPR signals for the ROSs were detected, which could account for the poor performance in organic contaminants removal during the O_2_ activation process, while the underlying mechanisms responsible for the limited generation of ROSs warrant further investigation. As for PTEB/O_2_/vis and PTEB‐NH_2_/O_2_/vis systems, both of the systems exhibited pronounced signals of DMPO‐O_2_^•−^ and TEMP‐^1^O_2_ adducts. However, relative weak TEMP‐^1^O_2_ and relative strong DMPO‐O_2_^•−^ signals were observed in PTEB‐NH_2_/O_2_/vis system when compare to the PTEB/O_2_/vis system, implying a divergence in the mechanisms of O_2_ activation and the predominant ROS in above two systems. Consequently, quantitative analysis of the contributions of ROSs was adopted to elucidate the predominant ROS in each system (Figure 3e; Table S4, Supporting Information). The results show that ^1^O_2_ is the dominant reactive species in the PTEB/O_2_/vis system, accounting for 56.3% of the ROSs generated through O_2_ activation, which indicats that the O_2_ activation on the pristine PTEB was more inclined to undergo the energy transfer pathway. In contrast, ^1^O_2_ accounts for only 35.3% and 23.4% of the ROSs in the PTEB‐CN/O_2_/vis and PTEB‐NH_2_/O_2_/vis systems, respectively. While O_2_^•−^ emerges as the predominant reactive specie, contributing 66.7% (PTEB‐CN) and 69.4% (PTEB‐NH_2_) to the total ROSs generated. This result is further proved by probe experiment using nitro blue tetrazolium (NBT), which can be solely reduced by O_2_^•−^.^[^ ^8^, ^41^ ^]^ Time‐resolved absorption spectra of NBT at 260 nm reveal that the O_2_^•−^ generation rates for PTEB‐NH_2_ and PTEB‐CN are 5.5 and 3.7 times higher than that of pristine PTEB, respectively (Figures 3f; S21 and Table S5, Supporting Information), emphasizing the significantly enhanced O_2_^•−^ production capability. The above experimental results demonstrate that the incorporation of –CN and –NH_2_ defects induces a fundamental shift in the primary ROS generated from O_2_ activation.

To further corroborate the function of charge carrier involved in the PTEB‐NH_2_/O_2_/vis system, quenching experiments employing AgNO_3_ and EDTA‐2Na as the scavengers photo‐generated e^−^ and h^+^ were conducted (Figure S20, Supporting Information). The addition of AgNO_3_ significantly inhibited the photocatalytic O_2_ activation performance, reducing the k_obs_ from 0.0883 to 0.0119 min^−1^. In contrast, the catalytic performance remained nearly unaffected upon the addition of EDTA‐2Na (k_obs_ switched from 0.0883 to 0.135 min^−1^), indicating that O_2_ activation on PTEB‐NH_2_ primarily relies on the reductive activation of photogenerated e^−^. This result was further confirmed by the EPR test conducted in the presence of BPA, N_2_, and AgNO_3_ (Figure 3g), which indicated that the generation of O_2_^•−^ in the PTEB‐NH_2_/O_2_/vis system originated from the electron‐involved O_2_ activation process.

### 2.5 IEF Dependent Charge Carrier Dynamics and Catalytic Mechanism

**Figure 4:**
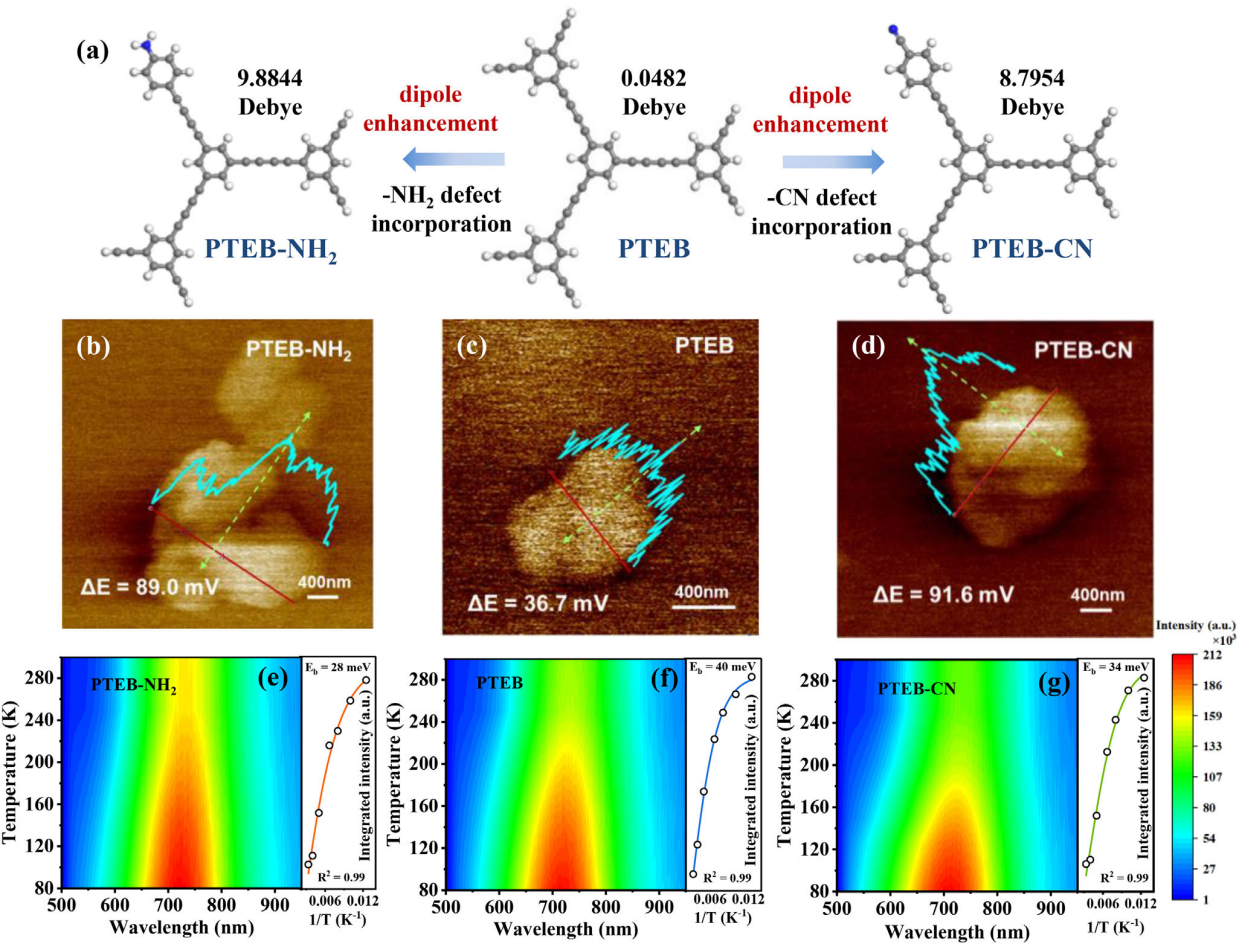
a) Terminal defect‐induced local dipole enhancement. b) The KPFM images and surficial electrostatic potentials of PTEB‐NH_2_ b), PTEB c), PTEB‐CN d). Temperature‐dependent PL spectra with a function of temperature of PTEB‐NH_2_ e), PTEB f), and PTEB‐CN g).

A key point affects the potocatalytic O_2_ activation and the reaction pathway orientation is the photo‐generated exciton dissociation process, which commonly hindered in the organic materials without sufficient internal driving force,^[^ ^10^ ^]^ as such, defect engineering could be a targeted strategy for tackling the occurred dilemma through manipulating the charge accumulation. Incorporation of –CN and –NH_2_ defects into the PTEB could induce alterations in the symmetry of the polymeric framework, which in turn affects the local dipole moments within the structure. Furthermore, the distinct electronegativity of these terminal defects substantially influences the electronic occupancy, collectively leading to significant electronic reallocation within the polymer structure. DFT calculation reveals that introduction of –CN and –NH_2_ defects could expand the local dipole from 0.0482 Debye (PTEB) to 8.7954 (PTEB‐CN) and 9.8844 (PTEB‐NH_2_) Debye (**Figure** **4**a), respectively, thereby increasing the local polarity of the polymeric structure, which is beneficial for improving the IEF. Based on the theory of Kanada model, the intensity of IEF could be caculated according to the Kelvin probe force microscopy (KPFM) (Figure 4b–d) and vector network analyzer (VNA) measurement (Figure S22, Supporting Information).^[^ ^9^ ^]^ It was observed that defect engineering could significantly enhance the IEF intensity of PTEB, with the IEF intensities for PTEB‐CN and PTEB‐NH_2_ determined to be 1.76 and 2.71 times greater than that of pristine PTEB, respectively (Figure S23, Supporting Information). With the enhanced IEF, sufficient internal driving force is established, allowing the generated excitons in the photocatalyst to dissociate into e^−^ and h^+^ under the influence of the IEF, leading to effective charge separation and subsequent transfer to the surface of the photocatalyst. Evidence for the enhanced IEF facilitating the separation of photogenerated electron‐hole pairs (e^−^/h^+^) can be provided by steady‐state photoluminescence (PL) spectra (Figure S24, Supporting Information). PTEB‐CN and PTEB‐NH_2_ displayed significantly diminished PL intensity, indicative of enhanced separation efficiency of charge carriers and a decrease in charge carrier recombination, suggesting that the IEF created by the ‐CN and –NH_2_ defects effectively modulates exciton behavior, thereby altering the reaction pathways between excitons and molecular O_2_. To further investigate the enhanced properties of PTEB‐CN and PTEB‐NH_2_, bi‐exponential functions were employed to fit the curves representing charge transfer dynamics, enabling quantitative analysis of the PL lifetime based on time‐resolved fluorescence decay spectra (Figure S25 and Table S6, Supporting Information). The incorporation of –CN and –NH_2_ defects significantly enhance the average decay lifetime (τ_avg_) of PTEB‐CN and PTEB‐NH_2_ (τ_avg_ = 2.59 and 2.63 ns, respectively) in compare with pristine PTEB (τ_avg_ = 1.98 ns), reflects the suppression of charge carrier recombination under the influence of the constructed IEF. In addition, exciton binding energy (E_b_) is a critical intrinsic parameter affecting excitonic effects of photocatalysts. Temperature‐dependent photoluminescence (TDPL) experiment over a broad temperature range (from 80 to 100 K) was conducted to calculate the E_b_ of the polymeric photocatalysts (Figure 4e–g; Table S7, Supporting Information):

(4) $$I\left(T\right)=\frac{I_{0}}{1+Ae^{-E_{b}/k_{B}T}}$$

where I(T) represents the integrated photoluminescence intensity at temperature T, measured on an energy scale; I_0_ is the integrated intensity at 0 K. A is a constant; k_b_ is the Boltzmann constant, 1.380649 × 10^−23^ J K^−1^. Based on the Arrhenius plots of the PL integrated intensities for the polymeric photocatalysts, the E_b_ for PTEB‐NH_2_, PTEB, and PTEB‐CN were determined to be 28, 40, and 34 meV, respectively. The remarkably decreased E_b_ values of PTEB‐NH_2_ and PTEB‐CN indicated the weakened strength of Coulomb interaction between the photo‐generated e^−^ and h^+^.^[^ ^42^ ^]^ As a result, the PTEB‐NH_2_ and PTEB‐CN possess higher local charge density, making the generated excitons more prone to dissociation and facilitating the generation of photogenerated charge carriers. While the generated excitons in PTEB encounter challenges in dissociation due to stronger exciton binding energy, resulting in their predominant activation of molecular O_2_ through an energy transfer pathway in the form of triplet excitons.

**Figure 5:**
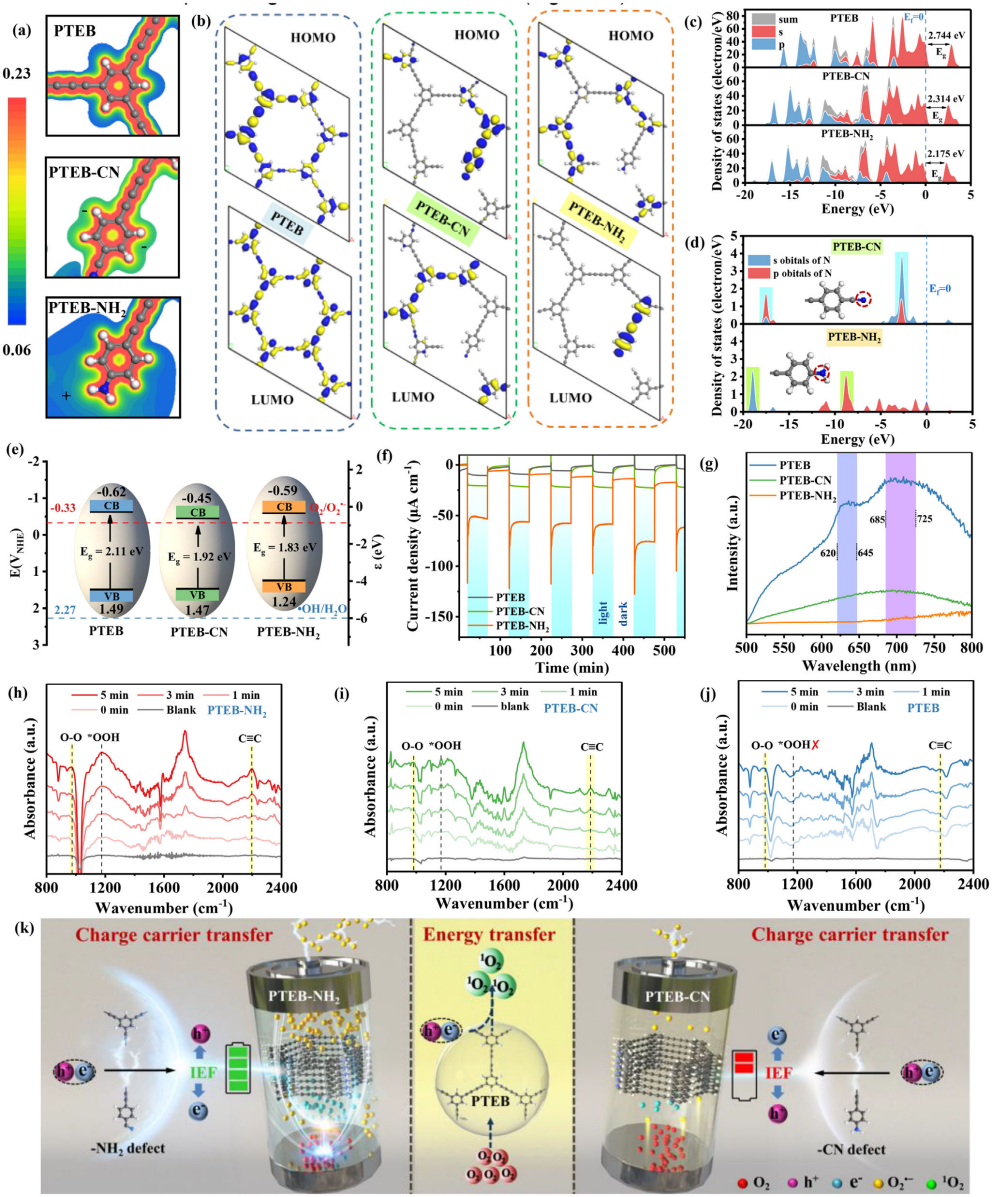
a) 2D slices of electrostatic potential distribution maps; b) charge density distribution of the HOMO and LUMO; c) calculated PDOS profiles; d) calculated PDOS of N element; e) bandgap and band position; f) transient photocurrents; g) Steady‐state phosphorescence spectra at 230 K (delay time = 1 ms); h–j) in situ DRIFTS spectra of PTEB, PTEB‐CN, and PTEB‐NH_2_. k) Schematic mechanism of the photocatalytic activation of molecular O_2_.

In addition, elucidating the behavior of e^−^ and h⁺ in PTEB‐NH_2_ and PTEB‐CN is essential for understanding their photocatalytic mechanisms. Electrostatic potential (ESP) slices (**Figure** **5**a) reveal that the –CN defect site is negatively charged with high electron density, while the ‐NH_2_ defect site is predominantly surrounded by electron clouds with low electron density. These observations suggest that the two types of defects induce distinct electronic redistribution behaviors, resulting in opposing directional trends in the constructed IEF. Orbital population analysis was employed to elucidate their separation dynamics under the constructed IEF. The highest occupied molecular orbital (HOMO) and lowest unoccupied molecular orbital (LUMO) locations of PTEB, PTEB‐CN, and PTEB‐NH_2_ are depicted in Figure 5b. For pristine PTEB, the HOMO and LUMO were uniformly distributed, which can be treated as the charge carriers evenly localized over the polymeric structure. Owing to the excitonic effect, the photogenerated e^−^ and h^+^ inclined to recombine,^[^ ^8^, ^43^ ^]^ leaving the handful of surviving carriers participating O_2_ activation reaction. In the case of PTEB‐CN and PTEB‐NH_2_, the spatial distribution of the HOMO and LUMO reveals a polarized arrangement owing to the driving force of IEF. Specifically, the HOMO of PTEB‐NH_2_ predominantly occupies the diacetylenic bonds and the linked three benzene rings that are situated away from the terminal defects, while the LUMO is primarily concentrated on the diacetylenic bonds adjacent to the ‐NH_2_ defect site. However, the orbital distribution was completely reversed in PTEB‐CN. Accordingly, the spatial separation of the photogenerated e^−^ and h^+^ is achieved in defective PTEB‐CN and PTEB‐NH_2_ through visible light irradiation. Due to the spatial hindrance between e^−^ and h^+^, carrier recombination is effectively suppressed. Therefore, O_2_ captured by PTEB‐CN and PTEB‐NH_2_ is more likely to be activated by charge carriers rather than by their bound excitonic states. Notably, since O_2_ can only accept e^−^ to get activated in the charge carrier transfer pathway, the –NH_2_ defects captured O_2_ can effectively utilize the surrounding enriched e^−^ to convert into O_2_^•−^. In contrast, O_2_ captured by the –CN defect faces a challenge to get activated due to the lack of sufficient e^−^.

In addition, analyses of the partial density of states (PDOS) of PTEB‐CN (Figure 5c,d) revealed that the maxima of the s orbitals and the p orbitals of the nitrogen element overlap, indicating that the defect energy level introduced by the ‐CN defects enters the total density of states (TDOS) of PTEB in the form of sp hybridization. However, the s orbitals of the nitrogen element introduced by –NH_2_ defects are located at a deeper energy level (energy<‐15 eV), which were difficult to utilize. Therefore, the defect energy level of PTEB‐NH_2_ is primarily composed of the p orbitals of the nitrogen element. The lower hybrid electron density of C atoms with adjacent N atoms in the VBM for the polymeric structure could reduce electrostatic attraction between the nuclei and electrons, making the photogenerated charge carriers easily transfer to the surface of the catalyst, stabilizing the charge carrier separation and inhibiting recombination. Moreover, The CBM and the VBM of PTEB, PTEB‐CN, and PTEB‐NH_2_ were mainly consists of C 2p states, while defect engineering introduced a defect energy level in the bandgap of the original PTEB due to the orbitals of nitrogen elements, which significantly reduces the bandgap energy (E_g_). This result is further proved by optical band structure with narrowed bandgaps (Figure S26, Supporting Information). According to the Tauc plot method (Figures S26 and S27, Supporting Information), the band structures of three PTEBs are displayed in Figure 5e. Since the CB positions of PTEBs are more negative than the standard O_2_/O_2_^•−^ redox potential (−0.33 V vs NHE),^[^ ^44^ ^]^ the generation of O_2_^•−^ via photo‐induced electron‐mediated O_2_ activation appears to be a viable pathway, which accounts for the existence of O_2_^•−^ in all the PTEBs/O_2_/vis systems. However, due to the overlap of HOMO and LUMO orbitals and the exciton effect induced by the strong E_b_ in PTEB, few free electrons can overcome Coulomb interactions to facilitate O_2_ activation. As a result, the PTEB/O_2_/vis system predominantly generates ^1^O_2_ as the primary ROS via the energy transfer pathway. Comparatively, the CB of PTEB‐CN (−0.45 V vs NHE) is only slightly more negative than the O_2_/O_2_^•−^ redox potential (−0.33 V vs NHE), indicating that the driving force for electron transfer from the CB of PTEB‐CN is less significant compared to that of PTEB‐NH_2_, which suggests that PTEB‐NH_2_ possesses a thermodynamic advantage over PTEB‐CN in facilitating O_2_ activation. This conclusion is further supported by the chronoamperometry experiments (Figure 5f) and electrochemical impedance spectroscopy (EIS) results (Figure S28, Supporting Information). A significant suppression of the recombination of photoexcited electron/ hole pairs is indicated by the increasing trend of transient photocurrent response (PTEB<PTEB‐CN<PTEB‐NH_2_). The photocurrent density of PTEB‐NH_2_ reaches ≈47.5 µA cm^−1^, significantly higher than that of PTEB‐CN (≈21.4 µA cm^−1^), suggesting that the nature of the –CN and –NH_2_ defects plays critical roles in modulating the exciton dynamics and thermodynamic behavior. Phosphorescence, arising from the emission of ISC‐generated triplet excitons, was also examined to elucidate the fate of excitons (Figure 5g). The pristine PTEB exhibits two phosphorescence emission peaks at 635 and 710 nm, in which the first emission peak could be attributed to the delayed fluorescence coming from the triplet excitons retransition back to the singlet state.^[^ ^45^ ^]^ In comparison, no significant phosphorescence emission peak was observed in PTEB‐NH_2_ and PTEB‐CN. The results suggest that the incorporated –NH_2_ and –CN defects alter the fate of generated excitons, shifting the fate of excitons from undergoing ISC to a triplet state toward dissociation into e^−^ and h^+^.

To better understand the distinct excitonic effects in PTEBs, their behaviors on O_2_ activation have been investigated using in situ diffuse reflectance FT‐IR spectroscopy (DRIFTS). Figure 5h–j presents the in situ DRIFTS spectra for O_2_ activation under a steam‐saturated O_2_ flow. The vibration signals at 971 cm^−1^ for all the PTEBs were attributed to the characteristic vibration of O─O originating from captured O_2_.^[^ ^46^ ^]^ The peaks corresponding to the stretching vibrations of diacetylenic bonds (C≡C, 2197 cm^−1^) in PTEBs exhibit a slight red shift (13 cm^−1^) upon exposure to the reaction mixture due to the adsorption of O_2_, indicating the pivotal role of C≡C as primary active sites accounting for O_2_ activation.^[^ ^47^ ^]^ The emerging peaks with progressively increasing signal intensity observed at 1173 cm^−1^ are attributed to the characteristic stretching mode of *OOH,^[^ ^28^ ^]^ a key intermediate in the charge carrier transfer pathway of O_2_ activation. However, this experimental evidence was exclusively observed in PTEB‐CN and PTEB‐NH_2_, while absent in pristine PTEB, which tell the mechanistic difference that pristine PTEB activates O_2_ solely through the energy transfer pathway, leading to the generation of ^1^O_2_, whereas the introduction of –CN and –NH_2_ defects enables a reaction pathway shift toward the charge carrier transfer pathway, facilitating the activation of O_2_ into O_2_^•−^. In addition, as the reaction progressed, the peak intensity corresponding to the intermediate *OOH in PTEB‐NH_2_ increased significantly more than that in PTEB‐CN, suggesting that the introduction of –NH_2_ defects is more effective in promoting *OOH formation compared to ─CN defects, which renders the captured O_2_ in PTEB‐NH_2_ more likely to undergo the charge carrier transfer pathway, reacting with photogenerated e‐ to achieve activation (Figure 5k).

### 2.6 Preliminary Scale‐up and Continuous Flow Experiment Design

**Figure 6:**
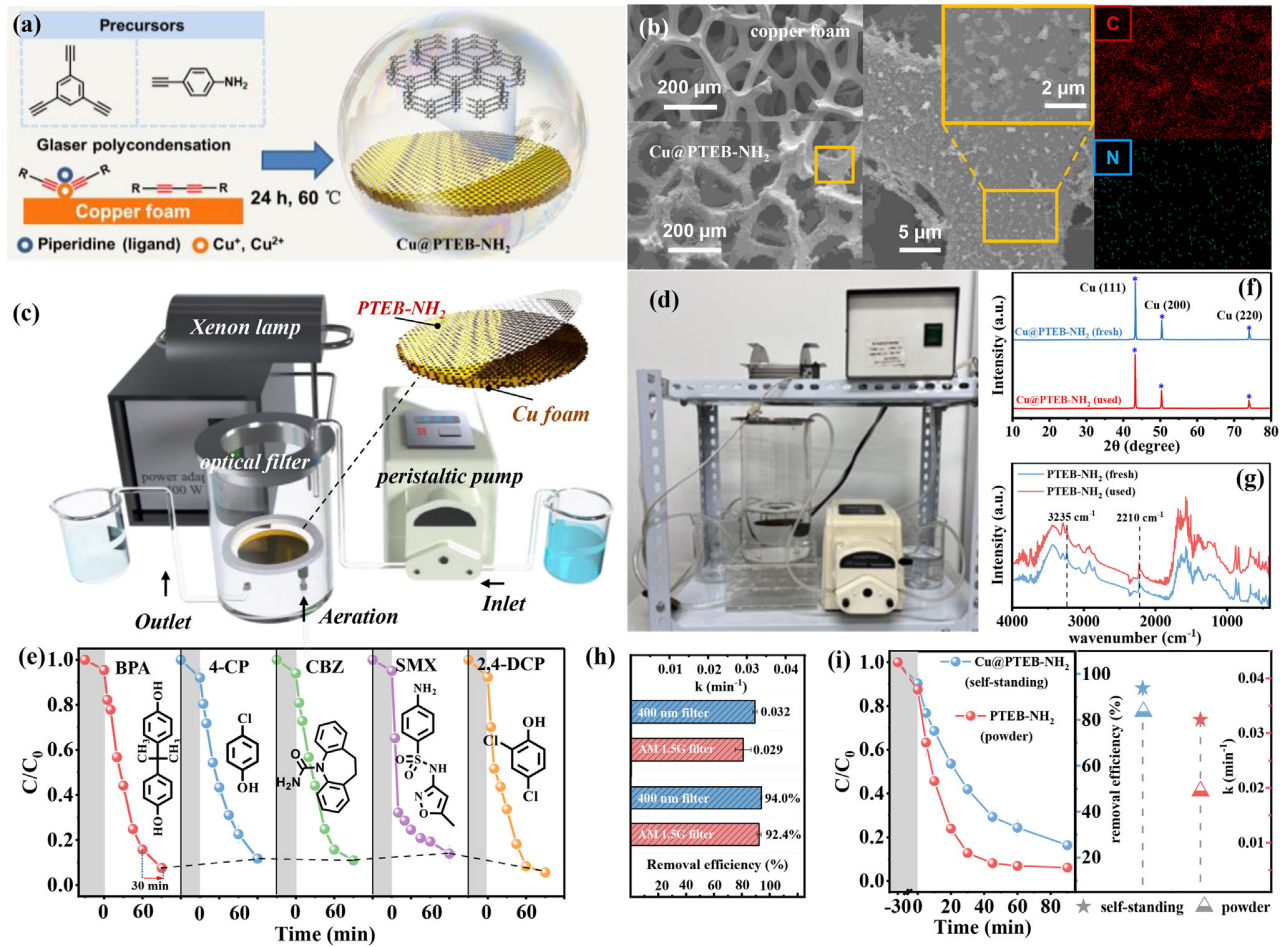
a) Schematic diagram of the synthesis procedure of Cu@PTEB‐NH_2_. b) SEM images of clean copper foam and Cu@PTEB‐NH_2_, as well as the EDS elemental mapping of synthesized Cu@PTEB‐NH_2_. Schematic flow c) and photograph d) of the continuous‐flow device. e) Removal performance of continuous‐flow device toward multiple contaminants within 90 min. FTIR spectra f) and XRD patterns g) of Cu@PTEB‐NH_2_ before and after use. h) Removal of BPA in a continuous‐flow device over different light resource. i) BPA removal efficiency over powdered PTEB‐NH_2_ and Cu@PTEB‐NH_2_ under solar irradiation.

For traditional photocatalyst‐mediated heterogeneous advanced oxidation processes (AOPs), two inherent drawbacks exist: 1) the dispersion of powdered photocatalysts within the reaction solution often results in a shielding effect caused by the upper‐layer catalysts, thereby impeding the efficient utilization of light energy; 2) recovering the photocatalyst from the reaction solution presents considerable challenges and is inevitably accompanied by photocatalyst loss.^[^ ^48^ ^]^ Fortunately, by leveraging the advantages of Glaser polycondensation, the polymerization of acetylenic monomers can be effectively achieved through a copper‐substrate‐catalyzed reaction to synthesize an immobilized catalyst, thereby overcoming the aforementioned limitations. As demonstrated in our previous work,^[^ ^18^ ^]^ a self‐standing polymers can be synthesized through the copper foam‐catalyzed polycondensation of terminal acetylenic groups (**Figure** **6**a, detailed synthesis procedures were provided in Supporting Information), enabling the in situ growth of PTEB‐NH_2_ (denoted as Cu@PTEB‐NH_2_, d = 10 cm). SEM images reveal that the amorphous PTEB‐NH_2_ polymer, characterized by localized protrusions, is uniformly coated on the surface of the copper foam while preserving its inherent porous structure (Figure 6b).

To ensure optimal visible light exposure for the photocatalyst, a circular Teflon sponge was utilized to support the photocatalyst. The continuous flow degradation tests were conducted by positioning the floating framework above the reaction solution (500 mL), with aeration applied from below to maintain dissolved oxygen saturation and ensure the uniformity of the reaction mixture. The entire reaction system operated in a quenching batch mode, with the hydraulic retention time (HRT) maintained at ≈90 min (Figure 6c,d). Control experiments first ruled out the contribution of Cu foam to contaminant removal (Figure S29, Supporting Information). Under visible light irradiation, the outlet analysis indicated that the BPA removal efficiency reached 91.3%. The feasibility of the Cu@PTEB‐NH_2_/O_2_/vis system can be conducted by employing other refractory contaminants as the target pollutant, including 4‐chlorophenol (4‐CP), carbamazepine (CBZ), sulfamethoxazole (SMX), and 2,4‐dichlorophenol (2,4‐DCP). The results demonstrate the robustness of Cu@PTEB‐NH_2_/O_2_/vis system as it could achieve over 85% removal ratio of the existing organic compounds (Figure 6e). The used Cu@PTEB‐NH_2_ was characterized by XRD and FTIR (Figure 6f,g), in which similar structural signal peaks were observed, indicating its structural stability. Replacing the 400 nm optical filter with an AM 1.5G optical filter to simulate solar irradiation, the self‐standing photocatalyst maintained a commendable contaminant removal efficiency (92.4%) and reaction kinetic (0.029 min^−1^) (Figures 6h; S30, Supporting Information), reflecting the Cu@PTEB‐NH_2_ substantial molecular O_2_ activation capability of Cu@PTEB‐NH_2_ under the driving force of solar irradiation. Thus, the as‐constructed continuous‐flow device was exposed to natural environmental conditions, utilizing natural sunlight as the light source instead of a laboratory‐based xenon lamp to evaluate the molecular O_2_ activation performance of Cu@PTEB‐NH_2_. For comparative analysis, we first employed powdered PTEB‐NH_2_ to evaluate the elimination performance of BPA under sunlight irradiation. As shown in Figure 6i, the powdered PTEB‐NH_2_ was able to eliminate ≈93.8% BPA within 90 min with a reaction constant of 0.0325 min^−1^. Comparatively, the O_2_ activation performance of Cu@PTEB‐NH_2_ was relatively less efficient, possibly due to the PTEB‐NH_2_ being immobilized on the solid Cu substrate, which reduced its contact area with molecular O_2_ and the reaction solution compared to powdered PTEB‐NH_2_. However, as Cu@PTEB‐NH_2_ overcomes the challenge of catalyst recovery commonly associated with powder catalysts, it significantly reduces catalyst loss and eliminates the need for additional recovery processes during application. Therefore, exploring its potential for large‐scale water purification has significant economic importance.

## 3 Conclusion

In summary, by analyzing the structure‐reactivity relation of a series of structural defects incorporated acetylenic polymer, PTEB‐CN and PTEB‐NH_2_ were screened out according to the guidance of Sabatier volcano correlation. The incorporated –CN and –NH_2_ defects manipulate the adsorption configurations, alter the thermodynamic barriers for key intermediate (*OOH) generation, as well as modulate the behavior of the involved excitons by constructing IEF during the pohotatalytic O_2_ activation. Specifically, defective PTEBs induce reversely oriented charge accumulation, which dictates the migration direction of charge carriers: hot e^−^ predominantly migrate through the conjugated framework toward the adjacent –NH_2_ defect sites (where O_2_ is adsorbed), while the –CN defect sites are primarily occupied by h⁺. This exciton‐oriented dissociation significantly weakens the exciton binding energy and switches the O_2_ activation pathway from non‐radical generation (via energy transfer) in pristine PTEB to selective O_2_^•−^ generation (via hot e^−^ transfer) in defective PTEBs. PTEB‐NH_2_ exhibits enhanced specific activity (100 mg L^−1^ g^−1^ h^−1^, PTEB‐NH_2_ > PTEB > PTEB‐CN) and O_2_^•−^ evolution selectivity (74.4%, PTEB‐NH_2_ > PTEB‐CN > PTEB), far exceeding other state‐of‐the‐art organic materials. Mechanism inquiry reveals that the incorporation of ‐NH_2_ defects has the capability to reduce the binding energy of hot excitons, facilitating the oriented migration of hot e^−^ toward adjacent acetylenic sites where O_2_ is adsorbed. Diverging from the process in pristine PTEB, where hot excitons generate ^1^O_2_ through the ISC process, this charge‐carrier transfer pathway not only alters the thermodynamics of ROS generation but also influences the subsequent kinetics of pollutant removal.

## 4 Experimental Section

### Preparation of PTEB

The synthesis of PTEB is based on copper species‐catalyzed Glaser polycondensation reaction. Initially, TEB (50 mg, 0.33 mmol) and copper(I) chlorid (CuCl 10 mg, 0.10 mmol) were dissolved in 10 mL pyridine and sonicated for 5 min to facilitate dissolution. Then the solution was sealed in a glass container and stirred for 24 h at 60 °C in a water bath. After polymerization, the product was collected by vacuum filtration, washed sequentially with pyridine, dichloromethane, methanol, and deionized water, and then subjected to vacuum freeze‐drying overnight to obtain the powdered PTEB.

### Preparation of PTEB‐CN and PTEB‐NH_2_

The synthesis of PTEB‐CN and PTEB‐NH_2_ was synthesized by a similar procedure except replacing a certain proportion of TEB with EBA and EBN. Specifically, for the synthesis of PTEB‐CN, the reaction mixture contained 43 mg of TEB (0.29 mmol, 7 equiv.) and 5 mg of EBN (0.04 mmol, 1 equiv.), while for the synthesis of PTEB‐NH_2_, the reaction mixture contained 45 mg of TEB (0.30 mmol, 7 equiv.) and 5 mg of EBA (0.043 mmol, 1 equiv.). After heating the reaction mixture in a 60 °C water bath with continuous stirring for 24 h, the defective PTEB‐CN and PTEB‐NH_2_ were obtained, followed by vacuum filtration and vacuum freeze‐drying to yield the product powder.

### Experimental Procedure and Analyses

The photocatalytic O_2_ activation performance of all of the samples was evaluated by the degradation of aromatic compounds under visible light irradiation. In a typical process, a catalyst (0.1 g L^−1^) was dispersed into bisphenol‐A (BPA) solution (0.1 mmol). The solution was continuously aerated with O_2_ and kept magnetically stirred under a 300 W Xenon lamp (65 mW cm^−2^) with a 400 nm cutoff. At each predetermined time interval, 1.0 mL of the reaction solution was acquired and filtered with a 0.45 µm water phase filter to separate the supernatant from the catalysts for analysis.

## Conflict of Interest

The authors declare no conflict of interest.

## Supporting information



Supporting Information

## Data Availability

The data that support the findings of this study are available from the corresponding author upon reasonable request.
