# Supplementary material for: Regulation of Excitonic Behavior in Defective Acetylenic Polymers Enables Mechanism Switching in O2 Activation for Enhanced Water Decontamination
Source: Adv Sci (Weinh). 2025 Dec 17;13(12):e11534. doi: 10.1002/advs.202511534 (PMC12948224; doi:10.1002/advs.202511534)
Supplement: Supplementary file 1 — Supporting Information [file ADVS-13-e11534-s001.docx]

**Supporting Information**

**Regulation of Excitonic Behavior in Defective Acetylenic Polymers Enables Mechanism Switching in O_2_ Activation for Enhanced Water Decontamination**

Xiaofeng Tang^[a,b]^, Sijia Jin^[a,b]^, Wei Li^[a,b]^, Yingrong Wang^[a]^, Haiyan Zhang^[b]^, Zhiqiao He^[a]^, Shuang Song^[a]^, Yaqi Cai^[b,c]^, and Tao Zeng*^[a,b,d]^

Tao Zeng*

Email: zengtao@ucas.ac.cn

**This PDF file includes:**

Supporting text

Figures S1 to S31

Tables S1 to S7

SI References

Supporting Information Text

Supplementary Methods

**Characterization.** The transmission electron microscopy (TEM) images were carried out on an accelerating voltage of 200 kV (Talos-S, FEI, USA). The scanning electron microscopy (SEM) images were obtained by a field-emission scanning electron microscope (Nano nova 450, FEI, USA) after spraying a thin gold layer. Energy-dispersive X-ray spectroscopy (EDX) was carried out using Genesis 4000 microscope with operating voltage 5 kV. The solid-state 13C magic-angle-spinning (MAS) NMR measurements were carried out using a Bruker Avance II solid-state NMR spectrometer (Bruker, Germany). Fourier transform infrared (FTIR) spectrophotometer obtained on a Thermo Scientific Nicolet iS20 (Thermo Scientific, America). X-ray diffraction (XRD) patterns were performed on a PANalytical X’ Pert PRO diffractometer using Cu Kα radiation (λ = 0.1541 nm). X-ray photoelectron spectroscopy (XPS) data were obtained on PerkinElmer PHI 5000 C instrument with a monochromatized Al Kα line source (200 W) The contact potential difference between the samples and the spectrometer, versus normal hydrogen electrode (NHE) at pH = 7, was estimated using the formula ENHE/V = Φ + VBmax - 4.44 (ENHE: potential of normal hydrogen electrode; Φ of 3.88 eV: the electron work function of the spectrometer). UV–Vis diffuse reflectance spectra (UV–Vis DRS) were performed on TU-1901 spectrophotometer equipped with diffuse reflectance accessories, using BaSO4 as the reference sample. (Shimadzu, Japan). The atomic force microscopy (AFM) and surface potentials were obtained via a Bruker Dimension Icon with a Kelvin probe. Photoluminescence (PL) spectra were recorded on an Edinburgh FLS1000 spectrophotometer. The electron paramagnetic resonance (EPR) measurements were carried out on a Bruker Model A300 spectrometer. Chronoamperometry, electrochemical impedance spectroscopy and linear sweep voltammetry performance were conducted in a conventional three electrode cell, using a Pt plate as the counter electrode and an Ag/AgCl electrode as the reference electrode. The working electrode was prepared on indium-tin oxide (ITO) glass. The 20 mg sample was dispersed in 300 μL of isopropanol and 50 μL Nafion by sonication to get slurry. The slurry was spread onto pretreated ITO glass. After air-drying, the Scotch tape was unstuck, and the uncoated part of the electrode was isolated with epoxy resin.

**Toxicity Prediction.** The acute and chronic toxicity of BPA and its intermediates were predicted utilizing the US EPA Ecological Structure Activity Relationships (ECOSAR) program. The risks associated with exposure to organic compounds for fish, Daphnia, and green algae were assessed using the ECOSAR software, employing quantitative structure-activity relationship models. The outcomes of the predictions encompass the half-maximum effective concentrations (EC_50_), half-maximum lethal concentrations (LC50), and chronic toxicity (ChV) values for each compound.

EC_50_ (half-maximum effective concentrations) is the concentration required to obtain a 50% of the maximum effect.

LC_50_ (half-maximum lethal concentrations) is the concentration required to kills 50% of the test creatures.

ChV (Chronic Value) is defined as the geometric mean of the no observed effect concentration (NOEC) and the lowest observed effect concentration (LOEC).

NOEC (No Observed Effect Concentration) is the highest tested concentration for which there are no statistical significant difference of effect (p<0.05) when compared to the control group in long-term ecotoxicity studies.

LOEC (Lowest Observed Effect Concentration) is the lowest concentration where an effect has been observed in chronic ecotoxicity studies.

**ROSs Quantitation Based on Quantitative EPR Measurements.**

The quantitative analysis of the contributions of ROSs was conducted based on the quantitative EPR measurements. First, to convert the raw first-derivative EPR signal into an absorption-type spectrum that is proportional to the radical concentration, and to eliminate the influence of factors such as line width and line shape, the EPR curve must be processed by double integration (DI). The resulting integrated area corresponds to the number of spins and can be expressed as Eq. 1:

$DI=\int_{\text{b}1}^{b2} dB\int_{b1}^{B} A(B')dB'=c[G_{R}C_{t}n]\frac{P^{1/2}B_{m}Qn_{B}S(S+1)n_{S}}{f(B_{1},B_{m})}$ (1)

where c = calibration constant;

G_R_ = receiver gain;

C_t_ = conversion time [s];

n = number of scans acquired;

P = microwave power [W];

B_1_ = microwave field [G];

B_m_ = modulation field [G];

Q = quality factor of resonator;

n_B_ = Boltzmann factor for temperature dependence;

S = total electron spin;

n_S_ = number of electron spins

f(B_1_,B_m_) = spatial distribution of the microwave and modulation fields as experienced by the sample.

When performing absolute spin quantitation to determine the ROS concentration in the target samples, several factors in Eq.1 can be cancelled out by taking the ratio of the DI for the target samples’s signal vs. that of the standard. Once the EPR spectrum for the standard and target samples have been collected, the number of spins in the target samples can be determine from the ratio of the signal intensities (DI) via Eq.2.

$n_{S}^{Un}=n_{S}^{St}\frac{DI^{Un}(P^{St})^{1/2}B_{m}^{St}}{DI^{St}(P^{U\text{n}})^{1/2}B_{m}^{Un}}$ (2)

Where the superscripts St and Un refer to the standard and unknown samples, respectively.

Computational details

**Structure.** The structures for PTEB, PTEB-CN and PTEB-NH_2_ were constructed according to the experimental procedures and the literature work^[1]^. A DMol3 method based on density functional theory was employed to geometrically optimize the proposed structures. DMol3 permits geometry optimisation and saddle point search with and without geometry constraints, as well as calculation of a variety of derived properties of the electronic configuration. (https://dmol3.web.psi.ch). DMol3 geometry optimized results can be used as a fast preoptimizer for further periodic DFT calculations.

**Adsorption energy.** The adsorption energy change (ΔE_ads_) of the key O_2_ activation intermediates, including *O_2_ and *OOH, was calculated relative to H_2_O and H_2_ under conditions of T = 298.15 K, pH = 0 and U = 0 V (vs. RHE)^[2]^.

**The Gibbs free energy variation.** Gibbs free energy change (ΔG) of each adsorbed intermediate was calculated based on the computational hydrogen electrode method developed by Nørskov et al9. At standard condition (T = 298.15 K, pH = 0, and U = 0 V (vs. RHE)), ΔG was defined as the following equation^[3]^:

ΔG = ΔE + ΔE_ZPE_ –TΔS + ΔG_pH_ + ΔGU

where ΔE was the energy change obtained from DFT calculation, ΔEZPE was the difference between the adsorbed state and gas phase, which was calculated by summing vibrational frequency for all model based in the above reaction system on the equation:

E_ZPE_ = 1/2∑hVi

T was the temperature = 298.15 K. ΔS represented the difference of the entropies between the adsorbed state and gas phase. The entropies of free molecules were obtained from NIST database (https://janaf.nist.gov/). ΔGpH = -kTln[H^+^] = pH·kT·ln10 = -0.0591pH was used to correct the free energy of H^+^ + e^–^ referenced by a RHE at various pH values. ΔGU = –neU, where U was the applied electrode potential and n was the number of transferring electrons. Hence, the equilibrium potential U_0_ for ORR at pH = 13 was determined to be 0.462 V (vs. RHE). The free energy of O_2_ (g) was derived as:

G_O2(g)_ = 2G_H2O(l)_ –2G_H2_ + 4.92 eV

Fig. S1. Atmotic structure of PTEB, PTEB-CN, PTEB-NH_2_, PTEB-F, PTEB-NO_2_ and PTEB-OH.

Fig. S2. The calculated E_ads_ for various photocatalyst-O_2_ configurations.


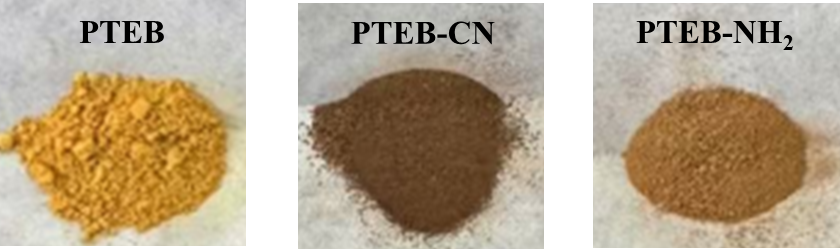


Fig. S3. Photograph of pristine PTEB, PTEB-CN and PTEB-NH_2_.


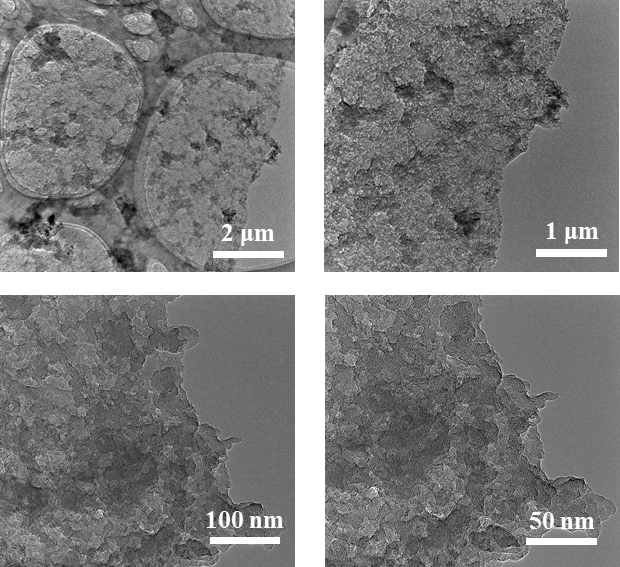


Fig. S4. TEM images of PTEB.


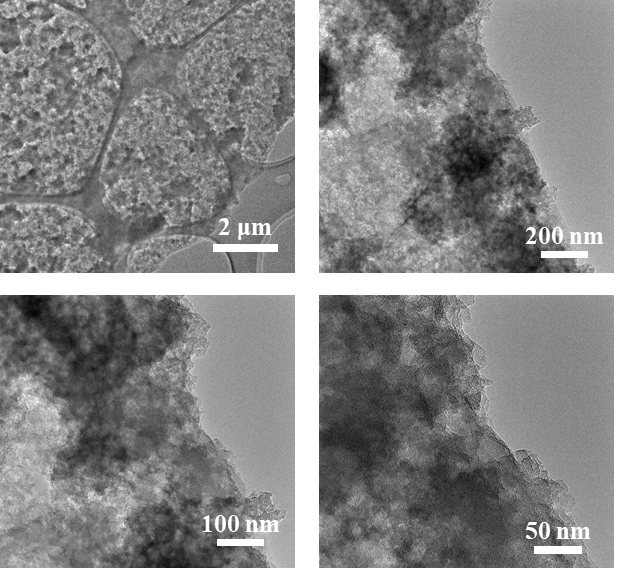


Fig. S5. TEM images of PTEB-CN.


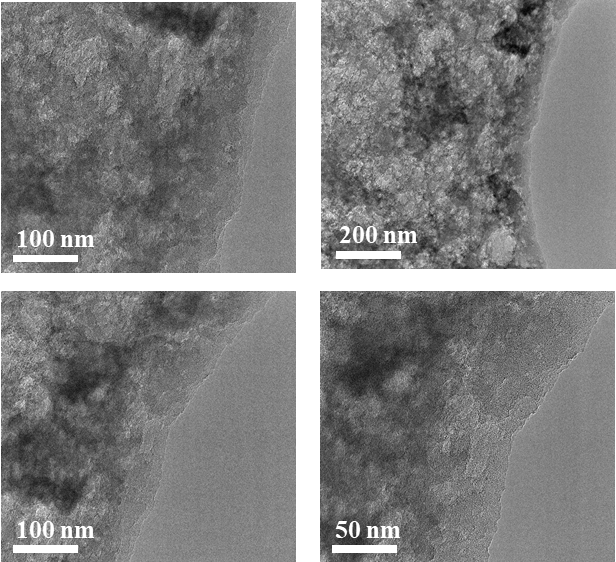


Fig. S6. TEM images of PTEB-NH_2_.


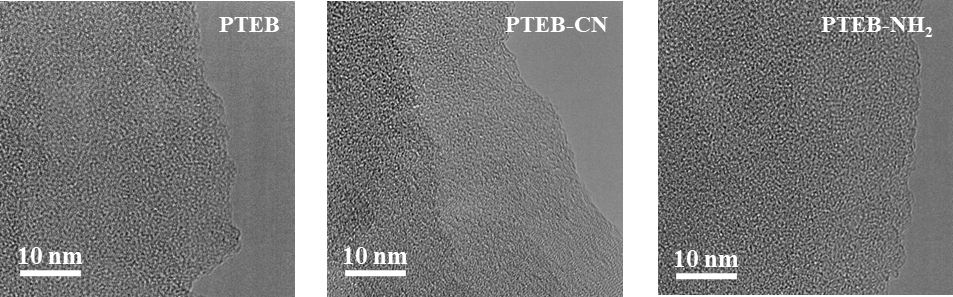


**Fig. S7.** HRTEM images of PTEB, PTEB-CN and PTEB-NH_2_.


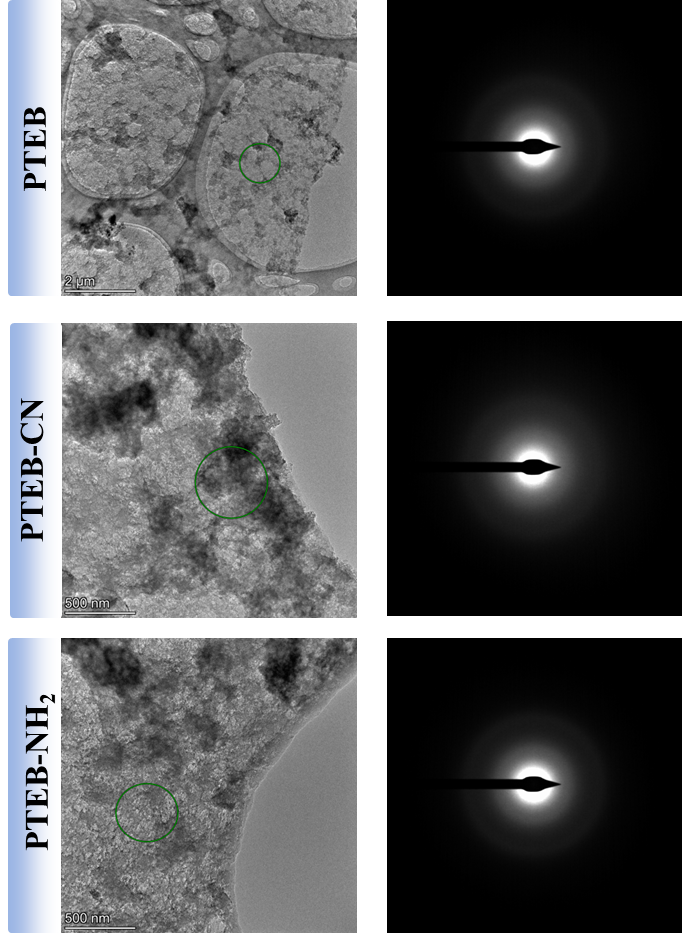


Fig. S8. TEM images and corresponding electron diffraction patterns of PTEB, PTEB-CN and PTEB-NH_2_ (the green cycles represent the selected area for electron diffraction).

Fig. S9. TEM and TEM-related EDS images of (a) PTEB and (b) PTEB-CN.


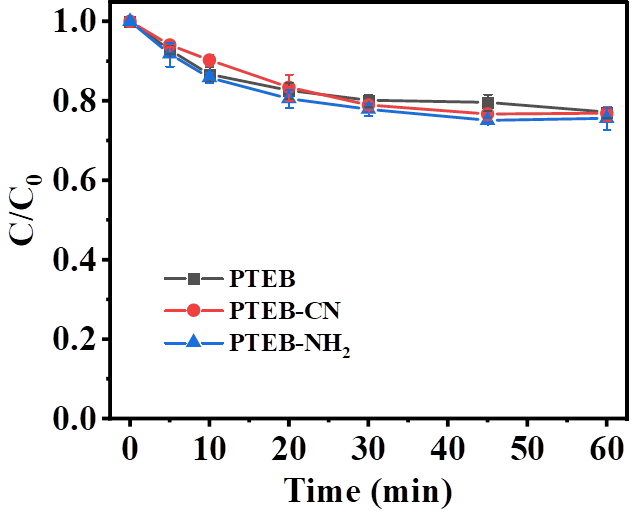


Fig. S10. BPA adsorption performance of PTEB, PTEB-CN and PTEN-NH_2_.

Fig. S11. The elimination of BPA in different reaction systems.

Fig. S12. The comparison of degradation performance based on photocatalytic O_2_ activation among PTEB-NH_2_ and other state-of-art photocatalysts.

Fig. S13. BPA degradation in PTEB-NH_2_/O_2_/vis system under different pH values.

Fig. S14. Effect of different anions (SO_4_^2-^, Cl^-^, CO_3_^2-^) on BPA removal efficiency in PTEB-NH_2_/O_2_/vis system.

Fig. S15. The TOC removal ratio in PTEB-NH_2_/O_2_/vis and PTEB-CN/O_2_/vis systems.

Fig. S16. LC/MS chromatogram and mass spectra for BPA in PTEB-NH_2_/O_2_/vis system. (a) Total ions chromatogram (TIC) from LC-MS; (b) mass spectra of the main peaks.

Fig. S17. Presentation of proposed degradation pathway of BPA in the PTEB-NH_2_/O_2_/vis system. Inset: Fukui index (f0) of BPA (The red triangle represents the sites in the BPA structure that are prone to attack by radical species).

Accordingly to the fukui function, the C_2_, C_4_, C_12_ and C_14_ atoms with the highest f^0^ value were vulnerable to radical attack by O_2_^•−^. The mineralization pathway of BPA was analyzed by conducting liquid chromatograph mass spectrometer (LC-MS) analysis (Figure S28). Based on the theoretical prediction of the attacking sites as well as the identification of intermediates, the main degradation pathways of BPA were proposed (Figure S29). The degradation of BPA was primarily attributed to two main pathways, namely, hydroxylation and β-scission. Owing to the C_2_, C_4_, C_12_ and C_14_ atoms with high f0 values of 0.47, the benzene rings of BPA could be first attacked by O_2_^•−^ to form P5 (m/z = 244), which could be further oxidized into P6 and underwent ring-opening reaction to form P7 (m/z = 216). The C_6_ and C_10_ atoms with relative high f- values of 0.031 were vulnerable to electrophilic attack of O_2_^•−^, leading to the β-scission of original BPA and transforming into P1 (m/z = 136) and P3 (m/z = 94). Meanwhile, P1 (m/z = 136) and P3 (m/z = 94) could be further oxidized to form two possible structures of ring-opened products P2 (m/z = 90) and P4 (m/z = 142). Finally, all of the hydroxylation and β-scission products underwent an aromatic ring opening process, leading to the formation of simple organic acids, which ultimately mineralized into CO_2_ and H_2_O.

Fig. S18. Toxicity analysis of BPA and its intermediates: acute toxicity LC_50_ of fish (a) and daphnid (b). Acute toxicity EC_50_ of green algae (c). Chronic toxicity Chv of fish (d), daphnid (e) and green algae (f).

According to the toxicity analysis based on ECOSAR system (Figure S30 and Table S7), the degradation intermediates demonstrated lower acute and chronic toxicity compared to the original BPA. Especially for intermediates P2 and P4, formed through the β-scission pathway, along with the more thoroughly oxidized products P8 and P9, exhibited ultrahigh LC/EC_50_ and ChV values, reaching a harmless level. The above results indicate that the PTEB-NH_2_/O_2_/visible light system can effectively reduce the biotoxicity of BPA.

Fig. S19. Effect of N_2_ purging during the degradation of BPA in the PTEB-NH_2_/O_2_/vis system.

Fig. S20. Effect of scavengers during the degradation of BPA in the (a) PTEB/vis system and (b) PTEB-NH_2_/O_2_/vis system. (c) Effect of SOD during the degradation of BPA in thePTEB-NH_2_/O_2_/vis system, [SOD] = 500 U mL^-1^.

Fig. S21. Photocatalytic NBT decomposition profiles of PTEB, PTEB-CN and PTEB-NH_2_. Routine condition: [NBT] = 0.01mM.

Fig. S22. Relative permittivity of PTEB, PTEB-CN and PTEB-NH_2_ measured by VNA.

Fig. S23. Internal electric field intensity of PTEB, PTEB-CN and PTEB-NH_2_.

Based on the theory of Kanada model, the intensity of IEF could be expressed as follows:

$$\text{E=}\sqrt{\frac{\text{-2}\text{V}_{\text{s}}\text{ρ}}{\text{ε}\text{ε}_{\text{0}}}}$$

Where V_s_ is the surficial electrostatic of the samples; ρ is the charge density; ε and ε_0_ refers to the dielectric constant of materials and the vacuum dielectric constant, respectively. Consequently, the surface potentials were measured by Kelvin probe force microscopy (KPFM) (Fig. 4b-d). The results show that the surficial electrostatic potential of PTEB-NH_2_ and PTEB-CN were 89.0 and 91.6 mV, respectively, which were 2.4 and 2.5 times the value of pristine PTEB (36.7 mV). The significantly enhanced surficial electrostatic potential of defective PTEB provides evidence that the artificial –NH_2_ and –CN defects were conducive to the improvement of electron density.^[4]^ A vector network analyzer (VNA) was utilized to measure the relative permittivity of the materials, resulting in average dielectric constants of 2.09, 2.07, and 2.12 for PTEB, PTEB-CN, and PTEB-NH_2_, respectively, over scanning frequencies (2-10 GHz) (Fig. S23), and the corresponding intensity of the IEF could be calculated accordingly.

Fig. S24. Photoluminescence spectra of PTEB, PTEB-CN and PTEB-NH_2_.

Fig. S25. Time-resolved fluorescence decay (b) spectra of PTEB, PTEB-CN and PTEB-NH_2_.

Fig. S26. (a) UV-vis diffuse reflectance spectra. (b) Tauc plots of PTEB, PTEB-CN and PTEB-NH2, the intersection of dash lines with x-axis indicates the individual bandgaps.

The calculation of optical bandgap was based on the Tauc plot method, which is based on the assumption that the energy-dependent absorption coefficient α can be expressed as:

$\left( \text{αhυ} \right)\text{1/}\text{n}\text{=}\text{A}\text{(}\text{hυ}\text{-}\text{E}\text{g}\text{)}$ (1)

where h is the Planck constant, υis the photon’s frequency, Eg is the bandgap energy, and A is a constant. The n factor depends upon the type of electron transition and equals 1/2 or 2 for direct and indirect transition bandgaps, respectively.[5] The calculated bandgaps in our work belong to the direct bandgaps (n=1/2). Therefore, the equation (1) can be expressed as:

$\left( \text{αhυ} \right)\text{2}\text{=}\text{A}\text{(}\text{hυ}\text{-}\text{E}\text{g}\text{)}$ (2)

Therefore, the optical bandgaps of samples can be obtained by extrapolation of the straight-line part of the Tauc plot ((αhυ)^2^ versus hυ graph).

As illustrated in Fig. S27a, compare with pristine PTEB with absorption edge at 415 nm, the absorption edge of PTEB-CN and PTEB-NH_2_ exhibit a red shift to 530 nm and 570 nm, respectively, resulting in a relative narrowed band gaps (1.92 and 1.83 eV) compare to pristine PTEB (2.11 eV) according to Tauc plot method (Fig. S27b).

Fig. S27. VB-XPS spectra for pristine PTEB, PTEB-CN and PTEB-NH_2_.

Fig. S28. Electrochemical impedance analyses spectra.

Fig. S29. The BPA adsorption performance test of Cu foam.

Fig. S30. BPA degradation in Cu@PTEB-NH_2_/O_2_/vis system under different light resources.

Table S1. The comparison of catalytic performance for organic catalysts in PMS activation.

| photocatalyst | catalyst loading  (g/L) | pollutant | Target Conc.  (mg/L) | η(t)%(min) | Removal Efficiency  mg L^-1^ g^-1^ h^-1^ | Ref |
| --- | --- | --- | --- | --- | --- | --- |
| PTEB-NH_2_ | 0.1 | BPA | 10 | ~99 (60) | 100 | This work |
| PTEB-CN | 0.1 | BPA | 10 | ~31 (60) | 31 | This work |
| PTEB | 0.1 | BPA | 10 | 52 (50) | 52 | This work |
| CC-UCN_2_ | 0.5 | BPA | 10 | ~100 (20) | 60 | ^[5]^ |
| BCN-680 | 0.4 | BPA | 20 | ~80 (30) | 80 | ^[6]^ |
| BDP-Por | 0.5 | BPA | 10 | ~99 (60) | 20 | ^[7]^ |
| PBNCZ-COO^-^ | 0.6 | BPA | 10 | ~99 (30) | 33 | ^[8]^ |
| Mn/S-C_3_N_4_ | 0.5 | BPA | 10 | ~99 (18) | 67 | ^[9]^ |
| CoSA-CN-20 | 1 | BPA | 10 | ~99 (60) | 10 | ^[10]^ |

Table S2. Fukui index of BPA

| Atom | f^0^ (BPA) | f^-^(BPA) | 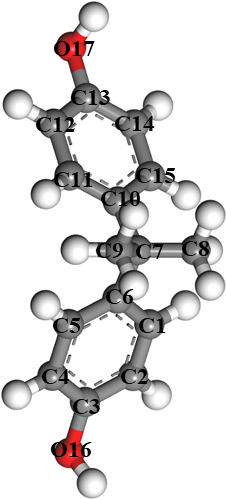 |
| --- | --- | --- | --- |
| C 1 | 0.027 | 0.025 |  |
| C 2 | 0.047 | 0.022 |  |
| C 3 | 0.023 | 0.050 |  |
| C 4 | 0.047 | 0.022 |  |
| C 5 | 0.027 | 0.025 |  |
| C 6 | 0.008 | 0.031 |  |
| C 7 | -0.016 | -0.012 |  |
| C 8 | -0.008 | -0.014 |  |
| C 9 | -0.008 | -0.013 |  |
| C 10 | 0.008 | 0.031 |  |
| C 11 | 0.027 | 0.025 |  |
| C 12 | 0.047 | 0.023 |  |
| C 13 | 0.023 | 0.050 |  |
| C 14 | 0.047 | 0.023 |  |
| C 15 | 0.027 | 0.025 |  |
| O 16 | 0.038 | 0.042 |  |
| O 17 | 0.038 | 0.042 |  |

Table S3. Acute toxicity and Chronic toxicity of BPA and its intermediates.

| Intermediates | Acute toxicity (mg·L^−1^) | | | Chronic toxicity (mg·L^−1^) | | |
| --- | --- | --- | --- | --- | --- | --- |
|  | Fish  (LC_50_) | Daphnid  (LC_50_) | Green algae  (EC_50_) | Fish  Chv | Daphnid  Chv | Green algae  Chv |
| BPA | 1.28 | 5.24 | 1.33 | 0.55 | 1.77 | 0.227 |
| P1 | 66.5 | 20.0 | 5.68 | 6.07 | 1.9 | 9.95 |
| P2 | 168000 | 67500 | 12100 | 10900 | 2520 | 1470 |
| P3 | 27.7 | 9.64 | 2.4 | 2.41 | 0.969 | 4.53 |
| P4 | 111000 | 51400 | 16500 | 8530 | 2850 | 2750 |
| P5 | 2.65 | 13.1 | 2.07 | 1.22 | 4.57 | 0.329 |
| P6 | 98.4 | 685 | 45.9 | 51.8 | 252 | 6.42 |
| P7 | 1.61 | 7.93 | 7.26 | 0.701 | 2.33 | 0.262 |
| P8 | 6130 | 2770 | 807 | 458 | 144 | 127 |
| P9 | 1710 | 946 | 629 | 162 | 85.4 | 155 |

Table S4. The detected ROSs concentration in systems composed of different photosensitizers.

| Photosensitizer | [•OH]  (mol/L) | [O_2_^•−^]  (mol/L) | [^1^O_2_]  (mol/L) |
| --- | --- | --- | --- |
| PTEB | 4.777×10^-6^ | 1.102×10^-5^ | 2.034×10^-5^ |
| PTEB-CN | 1.738×10^-6^ | 3.356×10^-6^ | 2.784×10^-6^ |
| PTEB-NH_2_ | 1.301×10^-6^ | 1.721×10^-5^ | 5.645×10^-6^ |

Table S5. Summary of the integrated peak area time-resolved absorption spectra of NBT during O_2_ activation.

|  | Integrated peak area | | |
| --- | --- | --- | --- |
| Time (min) | PTEB | PTEB-CN | PTEB-NH_2_ |
| 0 | 6.03541 | 6.73282 | 9.13314 |
| 5 | 5.13647 | 6.44472 | 4.07273 |
| 10 | 3.92147 | 5.91811 | 3.59512 |
| 20 | 3.3241 | 5.80277 | 3.27941 |
| 30 | 3.24216 | 5.67161 | 2.5671 |
| 45 | 2.366 | 4.96908 | 1.5834 |
| 60 | 1.6828 | 4.91335 | 1.2483 |

Table S6. Summary of the photoluminescence decay time (τ) and their relative amplitude (f) in PTEB, PTEB-CN and PTEB-NH_2_, which are derived from the steady-state PL spectra.

| catalysts | Decay time (ns) | | Relative amplitude (%) | | Mean (ns) |
| --- | --- | --- | --- | --- | --- |
|  | τ_1_ | τ_2_ | f_1_ | f_2_ | τ_avg_ |
| PTEB | 0.43 | 2.62 | 71.67 | 28.33 | 1.98 |
| PTEB-CN | 0.25 | 2.83 | 54.00 | 46.00 | 2.59 |
| PTEB-NH_2_ | 0.23 | 2.91 | 59.38 | 40.62 | 2.63 |

The average lifetime was calculated using equation: τ_avg_ = (f_1_τ_1_^2^ + f_2_τ_2_^2^)/ (f_1_τ_1_ +f_2_τ_2_)

Table S7. Summary of the average photoluminescence decay time in PTEB, PTEB-CN and PTEB-NH_2_, which are derived from the steady-state PL spectra.

| Time  (min) | Average decay time (ns) | | |
| --- | --- | --- | --- |
|  | PTEB | PTEB-CN | PTEB-NH_2_ |
| 0 | 6.03541 | 6.73282 | 9.13314 |
| 5 | 5.13647 | 6.44472 | 4.07273 |
| 10 | 3.92147 | 5.91811 | 3.59512 |
| 20 | 3.3241 | 5.80277 | 3.27941 |
| 30 | 3.24216 | 5.67161 | 2.5671 |
| 45 | 2.366 | 4.96908 | 1.5834 |
| 60 | 1.6828 | 4.91335 | 1.2483 |

**SI References**

[1]T. Zhang, Y. Hou, V. Dzhagan, Z. Liao, G. Chai, M. Loffler, D. Olianas, A. Milani, S. Xu, M. Tommasini, D. R. T. Zahn, Z. Zheng, E. Zschech, R. Jordan, X. Feng, *Nat Commun* **2018**, *9*, 1140; T. Zeng, X. Tang, X. Cai, S. Jin, Y. Zhu, W. Xu, S. Song, H. Zhang, *ACS Catalysis* **2024**, *14*, 1405-1418.

[2]W. Tang, E. Sanville, G. Henkelman, *J Phys Condens Matter* **2009**, *21*, 084204.

[3]J. K. Norskov, J. Rossmeisl, A. Logadottir, L. Lindqvist, J. R. Kitchin, T. Bligaard, H. Jonsson, *J Phys Chem B* **2004**, *108*, 17886-17892.

[4]M. Xu, R. Wang, H. Fu, Y. Shi, L. Ling, *Proc Natl Acad Sci U S A* **2024**, *121*, e2318787121.

[5]Q. Zhang, J. Chen, H. Che, B. Liu, Y. Ao, *Small* **2023**, *19*, e2302510.

[6]D. An, S. Zhao, Y. Zhou, J. Fang, S. Zhuo, *Chemical Engineering Journal* **2024**, *498*.

[7]D. Wang, W. Zhao, F. Tan, X. Wang, J. Dong, S. Zhou, G. Liu, P. Gu, *Advanced Functional Materials* **2024**, *35*.

[8]X. Xia, J. Feng, Z. Zhong, X. Yang, N. Li, D. Chen, Y. Li, Q. Xu, J. Lu, *Advanced Functional Materials* **2023**, *34*.

[9]C. Sui, Z. Nie, X. Xie, Y. Wang, L. Kong, S. Q. Ni, J. Zhan, *J Environ Sci (China)* **2025**, *149*, 512-523.

[10]X. Liu, Y. Zhang, P. Sun, F. He, Y. Wu, S. Wang, S. Wang, J. Zhang, *Angew Chem Int Ed Engl* **2025**, *64*, e202507028.
